# Antibody signatures in hospitalized hand, foot and mouth disease patients with acute enterovirus A71 infection

**DOI:** 10.1371/journal.ppat.1011420

**Published:** 2023-06-01

**Authors:** Lei You, Junbo Chen, Yibing Cheng, Yu Li, Yao-Qing Chen, Tianlei Ying, Lance Turtle, Hongjie Yu

**Affiliations:** 1 Savaid Medical School, University of Chinese Academy of Sciences, Beijing, China; 2 Shanghai Institute of Infectious Disease and Biosecurity, Fudan University, Shanghai, China; 3 Hospital Affiliated to Zhengzhou University, Henan Children’s Hospital, Zhengzhou, China; 4 Division of Infectious Diseases, Chinese Centre for Disease Control and Prevention, Beijing, China; 5 School of Public Health (Shenzhen), Sun Yat-sen University, Shenzhen, China; 6 School of Basic Medical Sciences, Fudan University, Shanghai, China; 7 NIHR Health Protection Research Unit in Emerging and Zoonotic Infections, Institute of Infection, Veterinary and Ecological Sciences, University of Liverpool, Liverpool, United Kingdom; 8 Liverpool University Hospitals NHS Foundation Trust, Liverpool, United Kingdom; 9 School of Public Health, Fudan University, Key Laboratory of Public Health Safety, Ministry of Education, Shanghai, China; University of Maryland, UNITED STATES

## Abstract

Enterovirus A71 (EV-A71) infection is a major cause of severe hand, foot and mouth disease (HFMD) in young children. The characteristics of EV-A71 neutralizing antibodies in HFMD patients are not well understood. In this study, we identified and cloned EV-A71-neutralizing antibodies by single cell RNA and B cell receptor sequencing of peripheral blood mononuclear cells. From 145 plasmablasts, we identified two IgG1 monoclonal antibodies (mAbs) and six IgM mAbs that neutralized EV-A71. Four of the IgM mAbs harbor germline variable sequences and neutralize EV-A71 potently. Two genetically similar IgM antibodies from two patients have recurrent heavy chain variable domain gene usage and similar complementarity-determining region 3 sequences. We mapped the residues of EV-A71 critical for neutralization through selection of virus variants resistant to antibody neutralization in the presence of neutralizing mAbs. The residues critical for neutralization are conserved among EV-A71 genotypes. Epitopes for the two genetically similar antibodies overlap with the SCARB2 binding site of EV-A71. We used escape variants to measure the epitope-specific antibody response in acute phase serum samples from EV-A71 infected HFMD patients. We found that these epitopes are immunogenic and contributed to the neutralizing antibody response against the virus. Our findings advance understanding of antibody response to EV-A71 infection in young children and have translational potential: the IgM mAbs could potentially be used for prevention or treatment of EV-A71 infections.

## Author summary

Hand, foot and mouth disease (HFMD) caused by enterovirus infections affects millions of young children annually. Enterovirus A71 (EV-A71) is a major causative pathogen of severe HFMD. EV-A71-specific antibody response is induced rapidly after illness onset, but the characteristics of primary antibody response to EV-A71 in young children are not well understood. Previous study demonstrated a dominant IgM producing EV-A71-specific antibody-secreting B cell response in children under 3 years old. However, no EV-A71-specific human IgM mAb has been reported. Here, we identified EV-A71 neutralizing mAbs, including two IgG1 and six IgM mAbs, from plasmablasts of EV-A71 infected children. IgM mAbs neutralize EV-A71 potently although possess few somatic mutations. We found sequence similarities between IgM antibodies from two patients. We selected EV-A71 variants in the presence of neutralizing mAbs and demonstrated that residues critical for neutralization are conserved among EV-A71 genotypes. We further explored epitope specific antibody responses in acute phase sera of EV-A71 infected patients and found that these neutralizing epitopes on the virus are immunogenic. Our findings advance understanding of antibody response in EV-A71 infected HFMD patients and can help inform the development of therapies against EV-A71 infections.

## Introduction

Hand, foot and mouth disease (HFMD) is an infection that commonly affects children caused by one of various enteroviruses, most commonly enterovirus A71 (EV-A71), coxsackievirus A16, and coxsackievirus A6 [1]. Outbreaks of HFMD have been reported in countries of the Western Pacific Region since the late 1990s [2]; HFMD epidemics have been occurring annually in China since 2007 [1]. HFMD is normally self-limiting; symptoms usually resolve 7 to 10 days after onset [2]. However, a proportion of the patients rapidly develop neurological and systemic complications that can be fatal [1, 3, 4]. The proportion of HFMD cases resulting in severe disease have been estimated to be 0.925% [95% CI 0.920–0.930%] for HFMD overall and 1.74% (1.72–1.75%) for disease due to EV-A71 [1]. Where the causative virus is laboratory confirmed, 93% of fatal HFMD cases are associated with EV-A71 infection [1].

EV-A71 belongs to the family *Picornaviridae*, genus *Enterovirus*, species *Enterovirus A*. It is a non-enveloped virus containing a single-stranded, sense-strand, polyadenylated RNA of approximately 7400 nucleotides [4]. EV-A71 capsid comprises 60 identical subunits, each of which contains a copy of the four structural viral proteins (VP1-VP4). EV-A71 is classified into genotypes based on the sequence of the gene encoding VP1; subgenotype C4a is dominant in mainland China [5]. Scavenger receptor class B, member 2 (SCARB2) is a major receptor for EV-A71, which mediates both virus attachment and uncoating [6]. Several other molecules, such as heparan sulfate proteoglycans (HS) and P-selectin glycoprotein ligand-1 (PSGL-1), enhance viral infection by attaching virus to the cell surface [6].

Humoral immunity is crucial for protection from EV-A71 infections. Maternal antibodies transferred across the placenta before birth and through breastfeeding may be protective against HFMD in young children [7]. Phase 3 clinical trials indicate that the efficacy of EV-A71 inactivated vaccines is >90% against EV-A71-related HFMD, and clearly show that humoral immunity correlates with protection [8–11]. No anti-viral therapy is available for the treatment of HFMD [3]. Studies in mouse models have showed that passive transfer of an EV-A71 neutralizing monoclonal antibody (mAb) protects against lethal EV-A71 infection: a single treatment of a mAb (CT11F9) within 3 days of infection conferred full protection [12]. Intravenous immunoglobulin (IVIg) has been widely used for treatment of severe EV-A71 infection [2]. However, IVIg could also contain non-neutralizing antibodies which may contribute to antibody dependent enhancement of EV-A71 infection [13]. Therapy using mAb selected for specific properties would be an improvement on IVIg treatment.

Antibody responses in HFMD patients with EV-A71 infections are not well understood. In the acute phase of EV-A71 infection, 80% of patients were found to have neutralizing antibodies against EV-A71 (≥ 1:8 antibody titer) one day after the onset of illness [14]. No correlation between neutralizing antibody titers and disease severity has been found in cohorts where disease has already developed [14], suggesting that further study on antibody responses is needed to understand the mechanism of protection. Antibody responses are distinct in children with different ages: EV-A71-specific antibody secreting cells in children aged ≥3 years produce IgG predominantly, but IgM is predominant in younger children [15].

Two publications have reported identification and characterization of mAb from EV-A71-infected children [16, 17]. Chen et al. identified four EV-A71 neutralizing antibodies by using a phage display library derived from peripheral blood mononuclear cells (PBMCs) of EV-A71-infected donors. However, the epitopes critical for neutralization of these antibodies were not studied [16]. Huang et al. sorted plasmablasts from three EV-A71 subgenotype B5 infected children, from which variable domain genes were amplified and cloned to produce mAbs. They characterized 12 representative neutralizing IgG mAbs and mapped the epitopes [17]. They did not use IgM and IgA plasmablasts to generate mAbs, and all subjects were ≥3 years [17]. Their findings indicate that these mAbs are derived from pre-existing memory B cells. They found some post-infection sera are not associated with any of the identified epitopes, indicating there should be other epitopes that remain to be determined, especially for EV-A71 subgenotype C4a, the most common circulating subtype in mainland China. EV-A71 infection is more severe in children aged 2 years and under, and severity decreases with increasing age [1]. More detailed characterization of the antibody response, including studying mAb of all isotypes in younger HFMD patients with primary infection with EV-A71 is needed. High throughput single-cell sequencing approaches have been used for efficient identification of antigen-specific antibodies [18].

In this study, we focused on the antibody response in young children during acute EV-A71 infection. This group has the highest risk of severe HFMD, but rarely has the antibody response been studied in this detail. We produced and characterized IgM and IgA mAbs in addition to IgG antibodies. Our objectives were to identify and characterize EV-A71 neutralizing mAbs, to map the epitopes on EV-A71 genotype C4 critical for neutralization, and to explore the epitope specific serologic response in young children during acute EV-A71 infection. We found potent neutralizing antibodies and determined the residues which were involved in the neutralization process. These residues are conserved among EV-A71 genotypes. Genetically similar neutralizing antibodies were identified from different patients, indicating the possible preferential usage of heavy chain variable gene segments in the initial response to EV-A71 infections in humans. Our results also indicate that these epitopes are immunogenic in EV-A71 infected HFMD patients.

## Results

### Antibody response in EV-A71 infected HFMD patients

Six children had hand, foot and mouth disease (HFMD) with laboratory-confirmed enterovirus A71 (EV-A71) infection were enrolled. The median age of participants was 25 months (range 7–35 months) (**S1 Table**). Three of them were severe HFMD patients who had central nervous system complications; the other three patients had mild HFMD without complication. All six patients recovered and discharged from hospital within 2 weeks. One PBMC sample was collected from each patient during hospitalization at one to five days after illness onset and was subjected to single-cell RNA sequencing (scRNA-seq) to obtain single-cell B cell receptor (BCR) sequences and gene expression data using the 10X Genomics Single Cell V(D)J Enrichment kit and Single Cell 5’ Library and Gel Bead kit. EV-A71 RNA is detectable by polymerase chain reaction in throat swabs or stool samples collected at the same day or one to two days after the collection of PBMC sample. It has been reported that EV-A71 viral load declines with increasing time after illness onset [19]. Thus, we conclude that EV-A71 was being shed in patients at the time of PBMC collection. The infecting EV-A71 strain in these patients belongs to subgenotype C4a.

Five of the six patients developed a neutralizing antibody response against EV-A71 at the time of PBMC sample collection (**S1 Fig**), thus they were selected for further analyses of antibody responses. Plasmablasts are rapidly induced during acute infections and contribute to early antibody responses [20, 21]. A previous study found that a robust plasmablast response specific to EV-A71 is detected in HFMD patients in the first week of illness [15]. Therefore, we selected plasmablasts from the five enrolled subjects for further production and screen of virus-specific antibodies. PBMCs were clustered based on the gene expression data. Cells in the B cell cluster expressing plasmablast signature genes MZB1, XBP1, and MKI67 were defined as plasmablasts (**S2 Fig**) [22]. A total of 228 plasmablasts with IgM, IgA, or IgG BCR variable gene sequences were recovered from scRNA sequencing data from the five subjects. IgM, IgA1, and IgG1 were the major immunoglobulin isotypes/subclasses that expressed in the plasmablasts in these acute phase samples (**S3A** and **S3B Fig**). No obvious association was found between antibody isotype/subclass and disease severity. The heavy chain variable gene usage of these plasmablast antibodies was analyzed using the IMGT/V-QUEST tool [23]. The variable gene usage of the heavy chain of these antibodies is biased toward IGHV3-IGHJ4 genes, especially in mild HFMD patients (**S3C** and **S3D Fig**).

### EV-A71 specific human monoclonal antibodies

**Fig 1:**
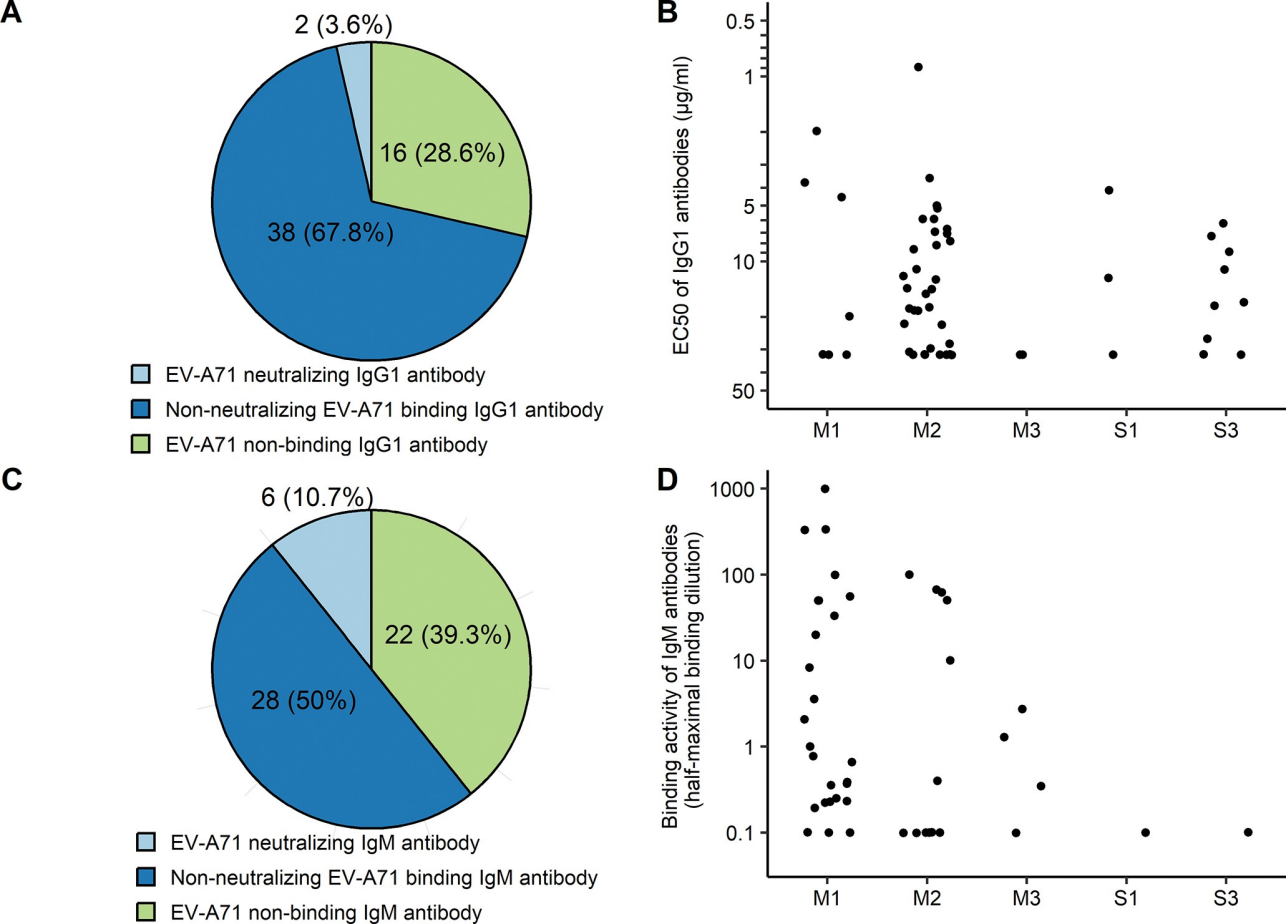
Characterization of IgG1 and IgM mAbs from EV-A71 infected children. (A) numbers and percentages of EV-A71 neutralizing IgG1 mAbs, non-neutralizing EV-A71 binding IgG1 mAbs and non-binding IgG1 mAbs. (B) EV-A71 binding activity of 56 IgG1 mAbs from the five study subjects were determined using ELISA. (C) numbers and percentages of EV-A71 neutralizing IgM mAbs, non-neutralizing EV-A71 binding IgM mAbs and non-binding IgM mAbs. (D) EV-A71 binding activity of 56 IgM mAbs were determined by ELISA using mAb containing culture supernatants. EC50 (half maximal effective concentration) values for IgG1 mAbs and half-maximal binding dilution of IgM mAb containing culture supernatants were calculated using GraphPad Prism 7.

We next sought to produce mAbs based on the BCR variable gene sequences of the plasmablasts. We firstly examined the BCR clonotype enrichment in the 228 plasmablasts. B cells that have the same variable gene usage and identical amino acids in the third complementarity determining region (CDR3) of both heavy and light chains were grouped into the same clonotypes. There are 201 unique clonotypes, including 78 IgG, 55 IgA, and 68 IgM. Among the 78 IgG clonotypes, 56 are IgG1. IgG1 is the predominant subclass of immunoglobulin in antiviral antibody responses [24]. Therefore, we firstly produced and purified the 56 IgG1 antibodies. The neutralizing and binding activity of the purified IgG1 mAbs with EV-A71 virions was determined by neutralization and ELISA assays. Forty out of 56 (71.4%) of these IgG1 mAbs are EV-A71 specific, as indicated by ELISA measurement of the binding of purified antibodies with EV-A71 virions (**Fig 1A and 1B** and **S2 Table**). Among the EV-A71 binding IgG1 antibodies, two neutralize EV-A71: M1-1 neutralizes C4b only, while M2-12 neutralizes both C4a and C4b (**Table 1**).

**Table 1:** EV-A71 neutralizing mAbs.

mAb	Isotype	V_H_	D_H_	J_H_	V_H_CDR3	Mutations (alterations)	κ/λ	V_L_	J_L_	V_L_CDR3	Mutations (alterations)	EC_50_(μg/ml) C4a	NT50 (μg/ml)^a^ C4a	NT50 (μg/ml)^a^ C4b
M1-1	IgG1	5–51*01	1–26*01	2*01	CARSPSWYFDLW	0 (0)	κ	1–39*01	1*01	CQQSYSTPQTF	0 (0)	1.97	Negative	8
M2-12	IgG1	3–30*03 or 18 or 3-30-5*01	3–10*01	3*01	CARVMYYFHSGDALEVW	11 (8)	κ	1–27*02	2*01	CQRYNSAPPYTF	5 (4)	0.89	4	16
M1-12	IgM	1–18*04	6–19*01	4*02	CARDVRGSGGWYPYYFDYW	3 (1)	κ	3–20*01	1*01	CQQYGSSPPTF	1 (1)	Not tested	Negative	64
M1-16	IgM	5-10-1*03	1–26*01	4*02	CARLISGSYYYW	2 (2)	λ	2–23*02	3*02	CCSYAGSSTVF	0 (0)	1.54	2	1.4
M1-17	IgM	4–4*02	3–10*01	6*02	CARVNYYGSGYYYYYGMDVW	0 (0)	κ	2–28*01	2*01	CMQALQTAYTF	0 (0)	0.7	0.25	0.25
M1-20	IgM	7-4-1*02	6–19*01	4*02	CARNLHVGLSGNFDYW	0 (0)	λ	3–1*01	2*01 or 3*01	CQAWDSSTVVF	0 (0)	0.12	0.3	0.5
M1-24	IgM	5–51*01	3–9*01	4*02	**CARQ***I*G-**ILTGYYPFDYW**^**b**^	0 (0)	κ	4–1*01	4*01	**CQQY**Y**S***T*P*L***TF**^**b**^	0 (0)	1.46	1.25	1.25
M3-7	IgM	5–51*01	3–9*01	4*02	**CARQ***Y*YD**ILTGYYYFDYW**^**b**^	0 (0)	κ	3–20*01	2*01	**CQQY**G**S***S*L*Y***TF**^**b**^	0 (0)	0.65	0.5	0.35

V_H_, variable gene segment of the heavy-chain variable domain; D_H_, diversity gene segment of the heavy-chain variable domain; J_H_, joining gene segment of the heavy-chain variable domain; Mutations (alterations), mutation number of variable domain nucleotides (amino acids); V_L_, variable gene segment of the light-chain variable domain; J_L_, joining gene segment of the light-chain variable domain.

^a^ Neutralizing antibody titer against EV-A71 subgenotype C4a/C4b.

^b^ Letters in bold: identical amino acids between M1-24 and M3-7, letters in italic: similar amino acids (similar chemical characteristics) between M1-24 and M3-7.

To produce IgA and IgM mAbs in addition to IgG that may have neutralizing activity against EV-A71, we used two further screening strategies at single-cell level, rather than cluster level, to select cells from the plasmablasts defined above (**S4 Fig**).

Firstly, we selected the expanded BCR clonotypes, because clonal expansion is a feature of activated B cells. Secondly, as opposed to using PBMC clustering, which is based on global gene expression, we instead selected cells that expressed the plasmablast signature genes XBP1, PRDM1, IRF4, and MKI67 at single cell level [20]. IgA and IgM antibodies from cells in the cluster of plasmablasts that met either one of these rules were selected and produced, including 28 IgA mAbs and 37 IgM mAbs.

The IgA/IgM containing HEK293F culture supernatants were used to measure binding and neutralization of EV-A71. Seven of the 28 (25%) IgA mAbs show weak binding activity with EV-A71 virion; none of them shows neutralizing activity. Twenty-five (67%) of the IgM mAbs are EV-A71 binding antibodies. Among them, we identified six (16%) EV-A71 neutralizing mAbs. M1-12 from donor M1 neutralizes C4b only, while M1-16, M1-17, M1-20, M1-24 from M1 and M3-7 from M3 neutralize both C4a and C4b (**Table 1**).

Because we did not find any neutralizing antibodies from the selected plasmablasts from patients with severe HFMD, and we found potent neutralizing antibodies from the selected plasmablasts from M1 and M3, we generated and screened further antibodies from plasmablasts of cases M1, M3 and S3, not using the two selecting strategies described above (**S4 Fig**). All antibodies from S1 had been screened before. We were able to make a further 13 IgM from M1, 6 IgM from M3, and 5 IgA antibodies from S3, from cells in the plasmablast cluster which were filtered out before by the two rules described above. Nine of the 19 IgM antibodies bind EV-A71, but no IgA mAb binds EV-A71. None of these 24 additional antibodies neutralizes EV-A71. Collectively, among the 56 IgM mAbs tested, six (10.7%) are neutralizing, while 28 (50%) are non-neutralizing EV-A71 binding antibodies (**Fig 1C and 1D**). In summary, eight EV-A71 neutralizing antibodies, including two IgG1 and six IgM antibodies, were identified from 145 BCR clonotypes of five HFMD patients (**S3 and S4 Tables**).

**Fig 2:**
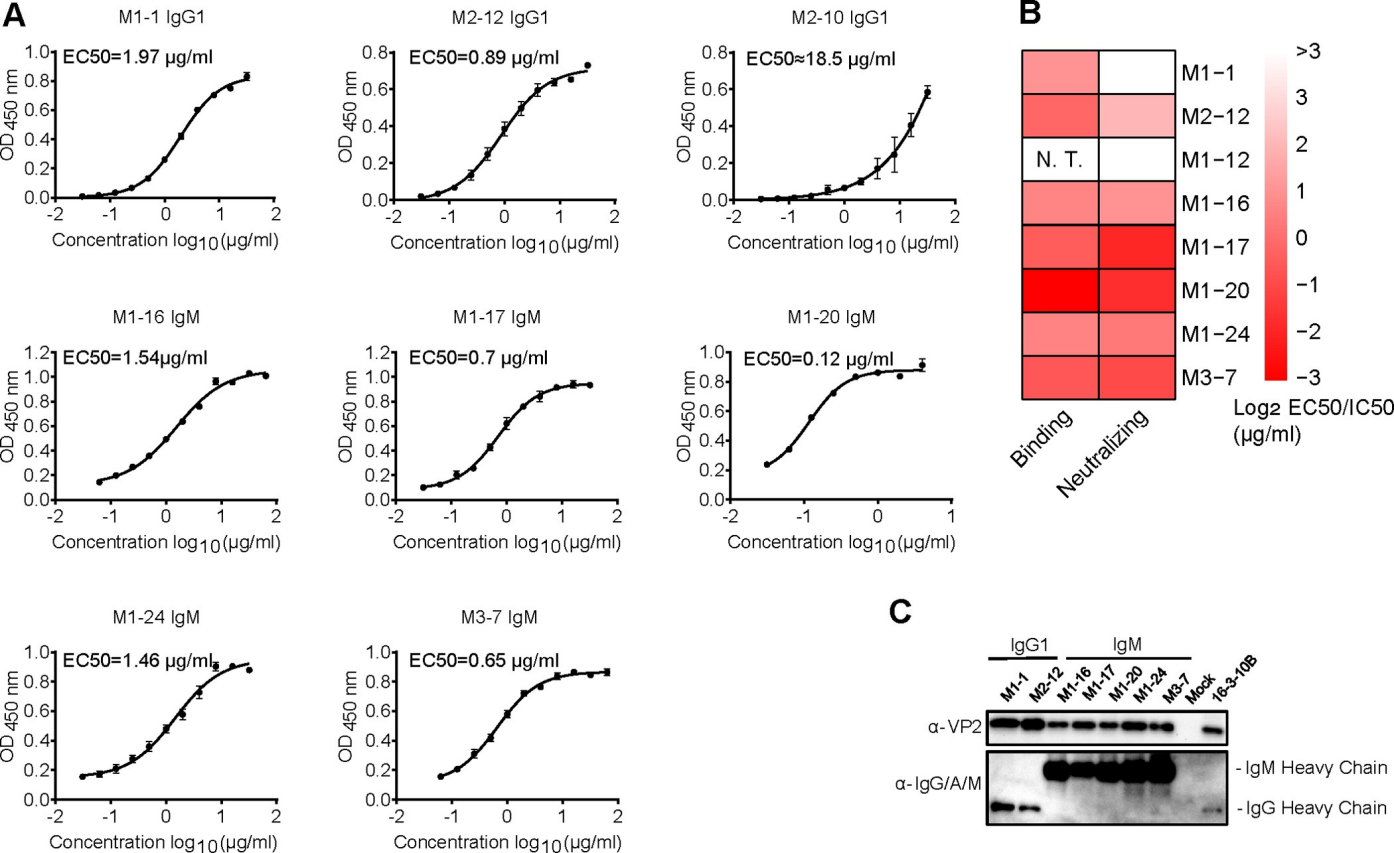
Binding activity of representative IgG1 and IgM mAbs with EV-A71. (A) ELISA was conducted using purified mAbs and mature EV-A71 subgenotype C4a virions; data shown are EV-A71 specific IgG1 and IgM mAbs and a poor binding IgG1 mAb (M2-10). The curves were fitted by nonlinear regression. (B) Heatmap showing ELISA and neutralizing antibody EC50 (half maximal effective concentration) values. (C) Immunoprecipitation of EV-A71 virions with mAbs to measure the binding of IgG1 and IgM antibodies to EV-A71. IgG mAb 16-3-10B was used as a positive control. 50% effective concentration (EC50) values were calculated using GraphPad Prism 7 software.

The neutralizing antibodies were purified and used to measure the binding and neutralizing titer. Binding of the neutralizing IgG1 and IgM mAbs with EV-A71 C4a virions was determined with ELISA assay and verified by immunoprecipitation assay (**Fig 2**). M1-12 was not included in these analyses as it does not neutralize C4a. Five of the IgM mAbs neutralize both C4a and C4b at similar concentrations, while the other one (M1-12) shows weak neutralizing activity against C4b but not against C4a (**Table 1**). EV-A71 specific mAbs were found in all five subjects; only the two severe HFMD patients not yielding any neutralizing mAb from plasmablast scRNA sequencing data.

### Heavy chain variable gene signatures in acute EV-A71 infections

**Fig 3:**
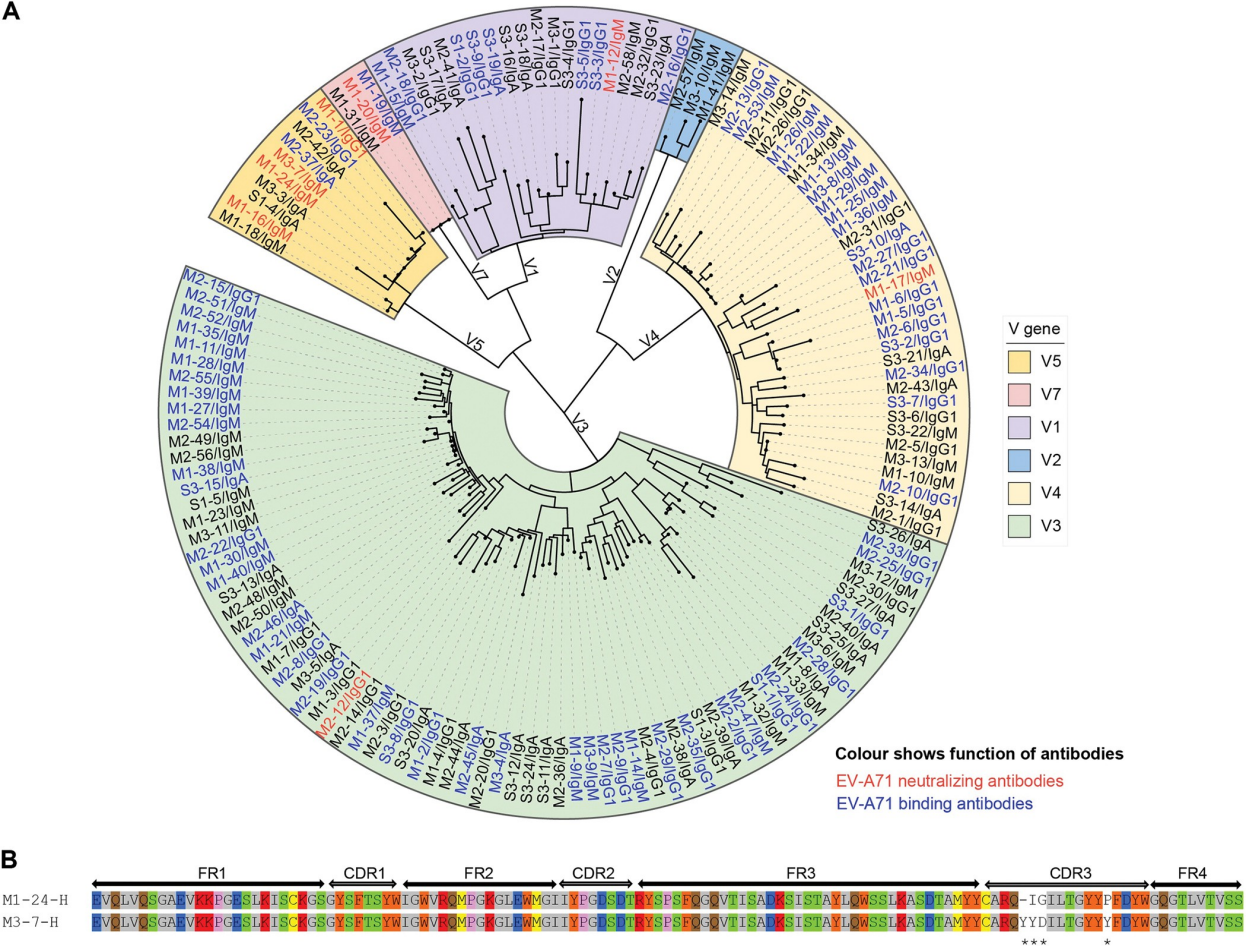
Heavy chain variable gene signatures in EV-A71 infected HFMD patients. (A) Maximum-likelihood phylogenetic tree of heavy chain variable region (V_H_) for all of the 145 antibodies screened was generated and arranged by IGH variable gene usage. Antibody ID and isotypes shown in bold red are EV-A71 neutralizing antibodies; those in blue are non-neutralizing EV-A71 binding antibodies; those in black are EV-A71 non-binding antibodies. The phylogenetic tree was generated by using MEGA 11. (B) Sequence alignment of heavy chain variable genes of M1-24 and M3-7. Amino acids are colored according to similar properties: blue, acidic; red, basic; green, hydroxyl; brown, amine; yellow, sulfhydryl; gray, aliphatic; orange, aromatic; pink, imino acid. Consensus across mAbs heavy chains is indicated below each amino acid residue by symbols: no symbol, conserved residue; asterisk, non-conserved residue.

A phylogenetic tree of heavy chain variable region was constructed, in which antibodies fall into groups by variable gene usage (**Fig 3A**). EV-A71-specific antibodies are found in five of the six heavy chain variable gene groups, except for V2 gene group. V5 genes are enriched in EV-A71 neutralizing antibodies, indicating biased variable gene usage (**S5 Fig**). Several antibodies from V1, V3, and V4 gene groups bind EV-A71, but most of them have no neutralizing activity. On the contrary, a higher proportion of EV-A71 binding antibodies using V5 genes are neutralizing antibodies.

We further analyzed the V_H_ gene usage for EV-A71 neutralizing antibodies, with six germline variable region gene segments identified from the IgG1 and IgM neutralizing mAbs. Among them, V_H_ 7-4-1 has been identified in EV-A71 neutralizing mAbs; V_H_ 3–30 and V_H_ 5-10-1 have been reported in non-neutralizing EV-A71 binding mAbs, while the other three have not been reported before. One of these three, V_H_ 5–51, was found in three mAbs from two subjects in this study (**Table 1**). Two neutralizing antibodies (M1-24 and M3-7) have the same heavy chain variable region gene segments and a V_H_CDR3 sequence identity of 83% (**Fig 3B** and **Table 1**), indicating a recurrent heavy chain variable region gene usage of antibodies induced by EV-A71 infection in different subjects, which has not been previously found in human antibodies induced by EV-A71 infection [17]. M1-24 and M3-7 use different light chain variable region genes. Recurrent variable region gene usage has been described in antibody response of other human virus infections, including SARS-CoV-2, influenza, and HIV [25–27]. One IgG1 mAb and two IgM mAbs harbor somatic mutations compared with germline variable domain sequences, while the other mAbs, including four potent EV-A71 neutralizing IgM mAbs (M1-17, M1-20, M1-24, and M3-7), share 100% sequence identity with the germline sequence (**Table 1**).

### mAbs neutralize EV-A71 at pre-attachment and post-attachment stages of infection

**Fig 4:**
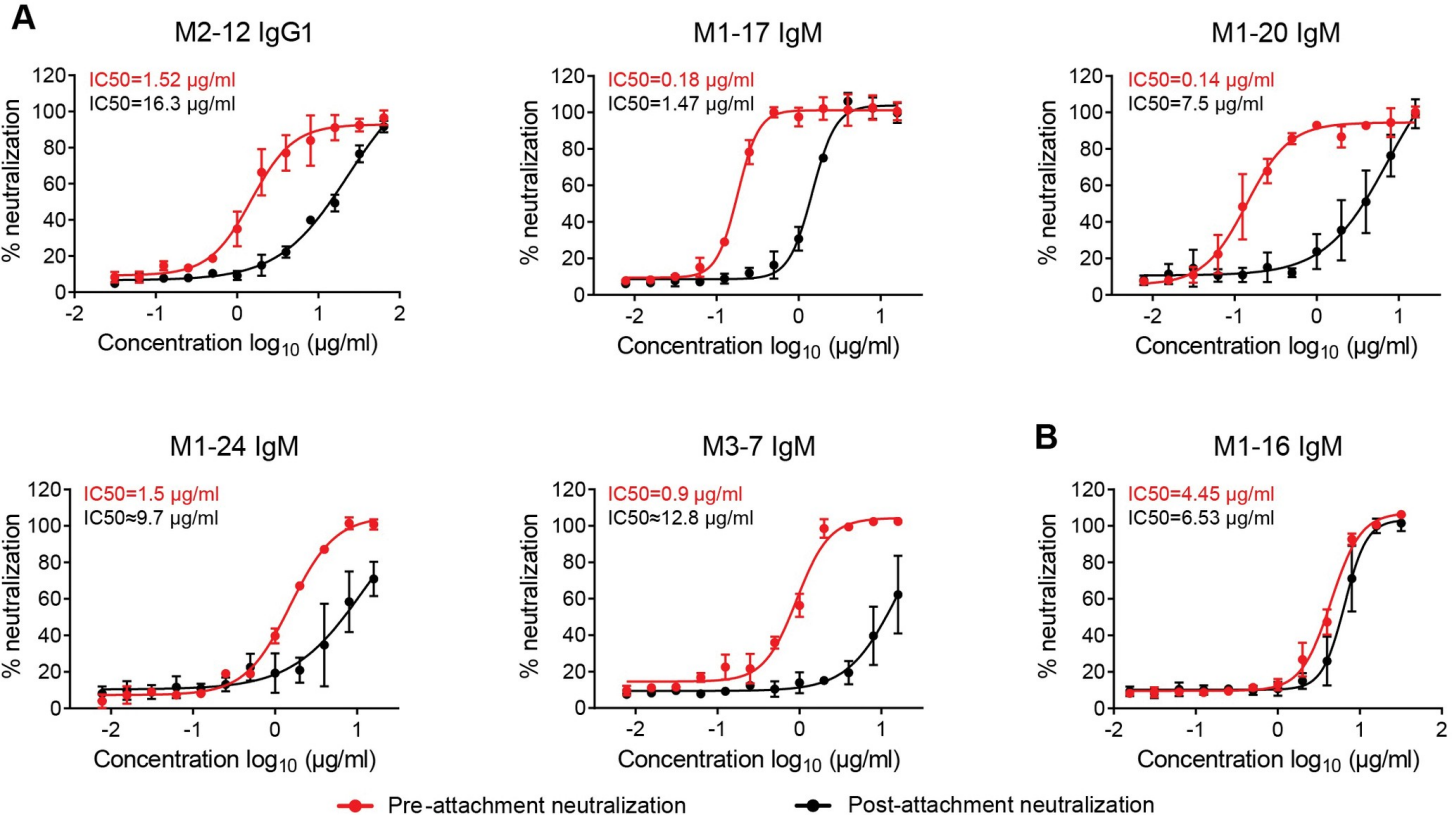
Neutralization of EV-A71 infection at pre-attachment and post-attachment stages by IgG1 and IgM mAbs. Inhibition of EV-A71 (C4a) infection at pre-attachment (red) and post-attachment (black) stages were determined by neutralization assay; cell viability was determined using Cell Counting Kit-8. (A) mAbs neutralize EV-A71 at post-attachment stage at much higher concentrations than at pre-attachment stage. (B) mAb neutralizes EV-A71 at similar concentrations at two stages. The curves were fit by nonlinear regression. Half-maximal inhibitory concentrations (IC50) were calculated using GraphPad Prism 7 software.

Antibodies with different neutralization mechanisms inhibit EV-A71 infection at different stages. Antibodies can neutralize EV-A71 by blocking receptor binding and inhibiting virus infection at the pre-attachment stage. Alternatively, EV-A71 can be neutralized by antibodies that block internalization or uncoating; such antibodies inhibit virus infection at both pre- and post-attachment stages [28, 29]. Neutralization can occur post attachment for other groups of viruses as well, such as flaviviruses [30] and coronaviruses [31]. We conducted pre- and post-attachment neutralization assays in parallel to explore the infection stages at which the antibodies neutralize EV-A71. For the post-attachment neutralization assay, the virus was incubated with plated cells at 4°C for 1 hour before adding serial dilutions of mAbs. An EV-A71 C4a strain was used and six mAbs neutralizing the strain, including one IgG1 and five IgM mAbs, were included for the analyses. As shown in **Fig 4**, five mAbs, M2-12, M1-17, M1-20, M1-24, and M3-7 inhibit EV-A71 infection at both pre-attachment and post-attachment stages with 8- to 53-fold higher concentrations of antibodies needed to inhibit EV-A71 infection at post-attachment stage compared with those for inhibition at pre-attachment stages. M1-16 inhibits EV-A71 infection at approximately similar concentrations at pre-attachment and post-attachment stages. Results of competition ELISAs for the EV-A71 neutralizing IgM antibodies using recombinant SCARB2 and PSGL1 show that M1-16, M1-17, M1-20, M1-24, M3-7 compete with SCARB2 for its binding with EV-A71, while only M1-16 competes with the binding of PSGL-1 (**S6C Fig**).

### Neutralizing epitopes on EV-A71

**Fig 5:**
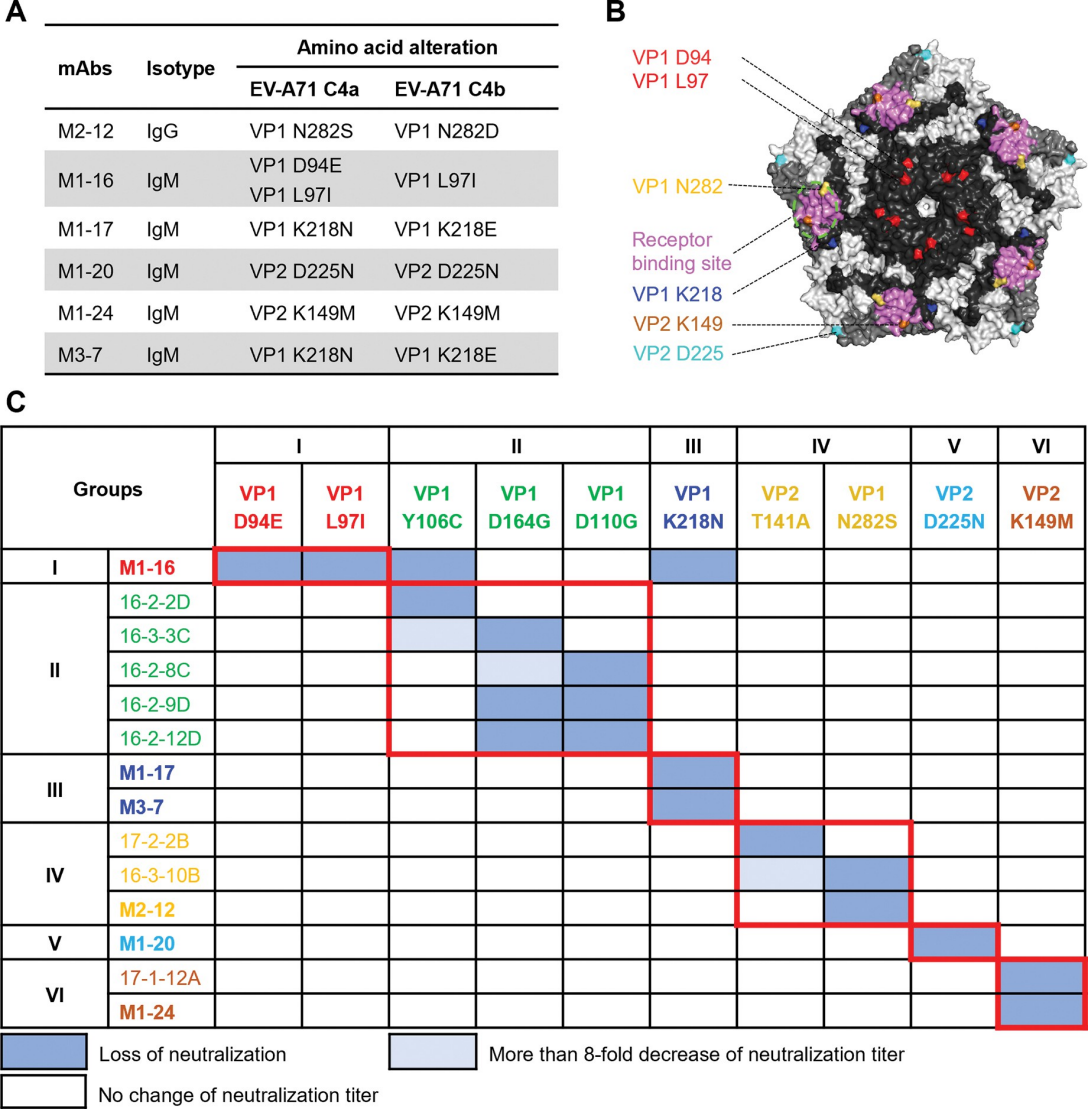
Epitopes on EV-A71 critical for neutralization. (A) EV-A71 Subgenotype C4a and C4b escape variants were selected by virus propagation in RD cells in the presence of excess mAbs. Escape variant strains were plaque purified and the P1 region was sequenced. We identified variants with substitutions at six positions on VP1 and VP2 proteins, which escape the neutralizing mAbs. (B) The residues critical for antibody neutralization and the SCARB2 binding site are mapped on EV-A71 virion surface. VP1 protein is colored in black, VP2 colored in gray, and VP3 colored in light gray. Residues critical for neutralization of M1-16 are colored in red; residue critical for M1-17 and M3-7 neutralization colored in blue; residue critical for M1-20 neutralization colored in cyan; residue critical for M1-24 neutralization colored in orange. The picture was created with PyMOL based on EV-A71 structure 3VBS [33]. (C) Cross-neutralization of mAbs, combining mAbs and escape variants of this study and those reported by Huang et al. [17], were determined by using wild-type and escape variant strains of EV-A71 C4a. Antibodies are grouped according to their cross-reactivity with the variants.

We further explored the epitopes on the EV-A71 particle that are involved in neutralization by selection of escape variants in the presence of antibody. One EV-A71 subgenotype C4a strain and one C4b strain were used for selection. M1-1 and M1-12 have no neutralization activity against C4a; thus, they were not used for selection of C4a escape variants. We failed to generate any C4b variant viruses that could escape M1-1 and M1-12; this may be due to the fact that these antibodies partially neutralize and are unable to fully prevent virus infection even at the highest concentration tested. As shown in **Fig 5A**, we successfully generated variants that escape neutralization of the other six antibodies. Each escaped virus contains an alteration at a single amino acid position on either the VP1 or VP2 proteins of EV-A71 (**Fig 5A**). These amino acid alterations confer resistance to neutralization of the corresponding mAbs. Under the selection pressure of antibody M1-16, we identified two escape variants derived from C4a, which both contain single amino acid substitution, VP1 D94E and VP1 L97I. On the other hand, only one C4b variant containing VP1 L97I substitution was generated in the presence of M1-16. VP2 K149, the residue critical for the neutralization of M1-24, is located in the receptor binding site of EV-A71 (**Fig 5B**) [32]. Another two residues, VP1 K218 and VP1 N282, are adjacent to the receptor binding site. All of the six residues identified here are conserved among EV-A71 genotypes (**S7 Fig**).

To cluster the epitopes critical for neutralization of human neutralizing antibodies against EV-A71, we tested the cross-reactivity among the mAbs and escape variants identified in this study and the neutralizing monoclonal antibodies and escape variants reported by Huang et al. [17]. The human IgG mAbs identified by Huang et al. were produced and used for selection of escape variants. We obtained escape variants that are consistent with those reported by Huang et al. (**S8A Fig**) [17]. We tested the cross reactivity of IgG and IgM mAbs and EV-A71 C4a escape variants using neutralization assays.

Neutralizing mAbs are grouped according to the loss of neutralization that occurs with each escape variant (**Fig 5C**). Epitope group I includes VP1 D94 and VP1 L97, which are located at the canyon northern rim and adjacent to the fivefold axis of EV-A71 particle (**S8B Fig**). Epitope group II contains VP1 Y106, VP1 D164, and VP1 D110 at the canyon floor. Epitope groups III and IV, which include residues VP1 K218 and VP2 T141, VP1 N282, respectively, are at the canyon southern rim. Epitope group V, which contains VP2 D225, is at the threefold axis of symmetry. Epitope group VI, which contains VP2 K149, is at the twofold axis of symmetry. Four residues, VP1 D94, VP1 L97, VP1 Y106, and VP1 K218, were found to be involved in the neutralization of M1-16: substitution of any one of the four abolishes the neutralization activity of M1-16. Three of the epitopes have not been reported to be associated with human antibodies before, including VP1 K218 critical for neutralizing of M1-16, M1-17, and M3-7, and VP1 D94E and VP1 L97 for M1-16.

### Epitope specific antibody response in EV-A71 infected HFMD patients

**Fig 6:**
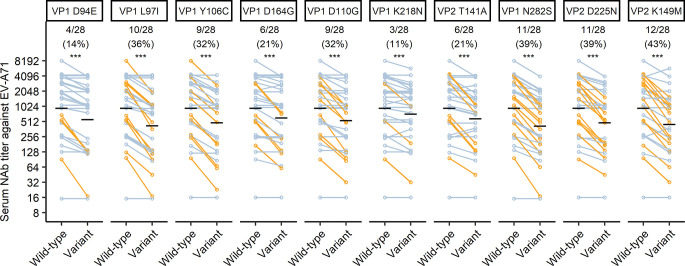
Epitope specific antibody response in EV-A71 infected HFMD patients. Neutralization titers against wild-type and escape variants of EV-A71 C4a in acute phase sera from 28 EV-A71 infected HFMD patients were tested. Serum samples showing a > 2-fold decrease in titer against variant(s) compared with wild-type were defined as sensitive to that variant(s) and results from these samples are highlighted in orange. Amino acid substitution in each variant and numbers of serum samples sensitive to each variant are shown above. Black lines show neutralization geometric mean titers of each column. The Wilcoxon signed rank test was used to compare log2 NAb titer against wild-type and variant strains for all samples tested (***: *p*<0.001).

To explore the role of these epitopes in neutralization of EV-A71 in HFMD patients, we measured the serum neutralizing antibody titer against wild-type and ten C4a escape variants in samples collected during the acute phase of EV-A71 infections from patients enrolled in the same cohort as patients selected for single-cell sequencing analyses. A total of 28 acute serum samples collected from 28 HFMD patients, including 17 severe and 11 mild patients, were included (**S5 table**). Neutralizing antibody titers against each variant strain from each patient’s serum were plotted in parallel with titers against wild-type strain and linked (**Fig 6)**. All samples tested neutralize wild-type EV-A71 strain and the ten variants, indicating that these sera are polyclonal and neutralize EV-A71 through multiple epitopes. The neutralization geometric mean titer against each variant is lower than that against wild-type strain, indicating that these epitopes are immunogenic and contributed to the neutralizing antibody response in EV-A71 infected children. Fold changes of the neutralization antibody titers against escape variants as compared with those against wild-type strain were calculated. An epitope was deemed to play a role in neutralization by a serum sample when the sample shows more than two-fold decrease in neutralizing titer against the corresponding escape variant strain as compared with wild-type EV-A71 C4a [34]. Data from samples which show >2-fold decrease in neutralization of the variant strain as compared with the wild-type are highlighted in orange. Four epitopes, including VP1 L97 (10/28), VP1 N282 (11/28), VP2 D225 (11/28), and VP2 K149 (12/28), are involved in neutralization by more than one-third of the serum samples.

The fold-change between neutralizing titer against wild-type strain and variants were compared between mild HFMD patients and severe HFMD patients; no significant difference was found for any of the variants (**S9 Fig**). We also explored the correlation between age of patients and fold change of neutralization titer against variant/wild-type strains. The results show a weak, but statistically significant, negative correlation for variant VP1 D94E (**S10A Fig**), in other words the component of serum neutralizing activity directed against VP1 D94 is greater with increasing age. No significant correlation was found for the other variants (**S10 Fig**).

## Discussion

In this study, we identified two IgG1 and six IgM monoclonal neutralizing antibodies from laboratory-confirmed EV-A71 infected HFMD patients. We found evidence of recurrent heavy chain variable region gene usage of neutralizing antibodies against EV-A71: heavy chains of two IgM antibodies from two subjects have the same variable region gene segments. Four of the IgM antibodies contain germline variable gene sequences and neutralize EV-A71 potently. Residues critical for neutralization by these antibodies are conserved among EV-A71 genotypes. The epitopes on EV-A71 critical for activity of human neutralizing mAbs fall into six groups. We also measured epitope specific neutralizing activity of acute phase serum samples from EV-A71 infected HFMD patients and found that the epitopes are immunogenic and contributed to the human neutralizing antibody response.

Antibody responses are induced rapidly after EV-A71 infection or vaccination [14]. EV-A71 specific IgM antibodies are positive in 90% HFMD patients in the first illness day [35]. In addition, the IgM antibody-secreting B cell response is dominant in children less than three-years-old [15]. Results of previous studies indicate that IgM might contribute to the neutralizing activity against EV-A71 at the early stages of infection, especially in young children. Consistent with these reports, we identified germline IgM mAbs with potent neutralizing activity, which might contribute to the fast induction of neutralizing antibody response after disease onset in EV-A71 infected HFMD patients.

IgM is the first antibody isotype produced by the host upon virus infection, meaning that it can serve as a biomarker for diagnosis of acute infection. In classical dogma, IgM is produced only during acute infection and is short-lived. However, in an animal model, influenza virus neutralizing IgM antibodies persist life-long (approximately 2 years) and protect against influenza virus challenge in mice [36, 37]. Neutralizing IgM in humans elicited by Zika virus persists for more than 1 year post infection and IgM is the dominant isotype contributing to neutralization in this period [38]. A protective role of IgM has been demonstrated against infection by various viruses in mice. Neutralizing IgM generated early in the course of infection plays a critical role in protection against West Nile virus, Chikungunya virus, and rabies virus [39–41]. Germline IgM is sufficient for control of influenza virus, Friend retrovirus and Sindbis virus infections [42–44]. In addition, germline IgM provided long-term protection against mouse hepatitis virus infection [45]. The presence of Japanese encephalitis virus-specific IgM is associated with survival in humans [46]. All of the four germline IgM mAbs identified in this study neutralize EV-A71 potently, indicating that the rapidly induced IgM antibodies in EV-A71 infected HFMD patients might confer protection against the pathogen.

IgM antibodies can be secreted into mucosal surfaces and constitute the first-line defense against mucosal pathogens [47]. The pentameric IgM interacts with pIgR expressed on the basolateral surface of epithelial cells and forms an IgM-pIgR complex that is transported across the cell to the mucosal surface [47]. The use of IVIg in the treatment of severe HFMD patients is empirical. It is challenging to standardize IVIg products, and no clinical trial data supports its usage. In addition, IVIg delivered intravenously may not reach the sites of virus infection and replication, which may limit its function. The potent IgM mAbs identified here could be a promising therapy for treatment of severe HFMD, with the caveat that they may not penetrate sanctuary sites like the brain. The germline nature of these mAbs implies that they would exhibit low immunogenicity and therefore high safety [48, 49]. In comparison with IgG antibodies, the prevention and treatment applications of IgM are understudied. One reason is that secreted IgM is pentameric, traditionally difficult to produce. Recent advances in manufacturing solve this problem [50]. Therapies using IgM antibodies in oncology are now in development [51]. The application of IgM mAbs, potent germline IgM mAbs in particular, in infectious diseases is promising. Intrarectal delivery of an IgM has been shown to reduce viral rectal infection of simian-human immunodeficiency virus in macaques [52]. Nasal delivery of an IgM offers broad protection from SARS-CoV-2 variants [50]. The potent neutralizing IgM mAbs identified in this study could potentially be used for prevention or treatment of EV-A71 infections.

Three groups of neutralizing epitopes critical for activity of human mAbs overlap with the binding site of SCARB2, the major receptor of EV-A71 [32]. SCARB2 supports attachment and internalization of EV-A71 and initiates conformational changes that lead to uncoating of viral RNA in the cytoplasm [6]. In addition to SCARB2, several other molecules that support cell surface binding of EV-A71 have also been identified [6]. HS and PSGL-1 are among the most studied attachment receptors. Both HS and PSGL-1 bind to positively charged residues close to the five-fold axis of EV-A71 virion, including VP1-145 [53, 54]. Alterations of EV-A71 VP1 protein at position 145 have been associated with disease severity [55–57]. VP1-145 G/Q/R are more frequently detected in severe human cases than VP1-145E. EV-A71 with VP1-145 G/Q/R binds HS and PSGL-1, while virus strains with VP1-145E do not. These data indicate that the binding of EV-A71 with HS and PSGL-1 might be associated with development of severe HFMD. Group I epitopes (VP1 D94 and VP1 L97) are close to the five-fold axis of EV-A71. Antibodies that bind to group I epitopes might inhibit infection of the virus by preventing its interaction with HS and thus potentially prevent the development of severe HFMD.

Recurrent variable region gene usage of human antibodies has been found for several pathogens but not EV-A71 before [25–27]. We identified two genetically similar EV-A71 neutralizing IgM antibodies from two patients, M1-24 from patient M1 and M3-7 from patient M3. These two antibodies use the same IGH variable region genes and similar V_H_CDR3 sequences. The mechanism of neutralization might be slightly different for M1-24 and M3-7 as different escape variants were identified. The difference in neutralizing mechanism might also be due to the differences in light chain sequences or lengths of V_H_CDR3 sequences. However, the key residues critical for neutralizing are close to each other, and overlap with the binding site of the EV-A71 receptor SCARB2. We also found a biased V_H_ gene usage for EV-A71 neutralizing antibodies as compared with binding antibodies. We speculate that these results are consistent with the possibility that humans preferentially use these gene segments to generate the initial response to EV-A71 infection. This hypothesis remains to be confirmed in further studies.

Our study has several limitations. First, no neutralizing antibody was identified from severe HFMD patients. We included two severe patients in the screening of EV-A71 specific antibodies and identified some antibodies with EV-A71 binding activity, including two IgG1 antibody from S1, seven IgG1 and three IgA antibodies from S3. None of them have a clinical history of an immune compromising condition. Second, we did not measure the EV-A71 binding concentration of non-neutralizing IgA and IgM antibodies. The purification of IgA and IgM antibodies are still much more complicated than that of IgG, so we only purified those with neutralizing activity. Third, only two genetically similar antibodies were identified. There might be more antibodies of this class against EV-A71 to be explored; our findings are only the beginning in this area. Fourth, the structural mechanism of antibody neutralization, and fifth, the role of non-neutralizing binding antibodies in protection remains to be explored in future studies. Finally, no children with asymptomatic EV-A71 infections were included in our study as acute phase samples were unavailable from them; thus, our data may not represent the full collection of antibodies induced by EV-A71 infection.

HFMD caused by enterovirus infection remains a major pediatric disease across the Western Pacific Region. Young children with primary EV-A71 infections have the highest risk of severe HFMD [1]. We found novel signatures of the antibody response to EV-A71 in young children hospitalized with HFMD. Our results demonstrate that IgM antibodies neutralize EV-A71 potently and show characteristics of recurrent variable gene usage. These findings advance our understanding of the primary humoral response to EV-A71 infection in young children. The potent neutralizing antibodies we identified represent potential therapeutic and prophylactic interventions.

## Materials and methods

### Ethics statement

The study was approved by the Institutional Review Boards of School of Public Health, Fudan University (IRB#2017-12-0654) and Henan Children’s Hospital (IRB#YZ-17-006). Written informed consent was obtained from parents or legal guardians of study participants on enrolment.

### Study subjects and samples

HFMD inpatients admitted to department of infectious diseases and pediatric Intensive Care Unit (ICU) of Henan Children’s Hospital between February 15, 2017, and February 15, 2018, were recruited as previously described [58]. Between February 15, 2018, and February 15, 2019, we continued to enroll severe HFMD patients who were admitted to ICU. Demographic information and clinical data during hospitalization were collected using standardized forms. Throat swabs and serum samples were collected within 48 hours of admission, peripheral blood mononuclear cells (PBMC) and stool samples were also collected during hospitalization. PBMCs were isolated by density gradient centrifugation and washed twice in PBS. PBMCs were cryopreserved in 10% DMSO in FBS in liquid nitrogen and thawed prior to use. Serum was isolated by centrifugation of serum tubes and aliquots were stored at -80°C. HFMD patients were classified according to the World Health Organization criteria [2]: 1) mild HFMD patients were patients had neither central nervous system nor systemic complications; 2) HFMD patients who had central nervous system complications such as brainstem encephalitis, encephalitis, encephalomyelitis or systemic complications such as pulmonary oedema/haemorrhage or cardiorespiratory failure were defined as severe HFMD patients.

### Single cell RNA sequencing and data processing

PBMC suspensions were barcoded through the 10x Chromium Single Cell platform. The scRNA-seq libraries were constructed by using the 5’ Library Kit, and the single-cell BCR sequencing libraries were constructed by using the V(D)J Enrichment Kits, Human B Cell. The libraries were sequenced using a next-generation sequencing platform. Single-cell sequencing data were aligned and quantified using kallisto/bustools (KB, v0.24.4) against the GRCh38 human reference genome. A neighborhood graph was built and used to calculate a Uniform Manifold Approximation and Projection (UMAP). Clusters were annotated based on the expression of canonical marker genes. B cell subsets were then selected, sub-clustered and annotated.

### Production and purification of mAbs

Heavy- and light-chain variable domain genes (**S4 Table**) were cloned into expression vectors and transfected into HEK-293F cells (ThermoFisher Scientific, R79007) using polyethylenimine (Polysciences, Inc. 23966) and cultured in serum-free expression medium (SinoBiological, M293TII) [59]. For production of dimeric IgA (dIgA) and pentamer IgM, the heavy and light chain plasmids were co-transfected with plasmid expressing the connecting J chain. Cell culture supernatant was collected 5 days after transfection and then assayed to determine neutralizing activity. IgG mAbs were purified by using protein A beads (Smart-Lifesciences, SA015025) and then assayed to determine antigen binding and neutralization. IgA and IgM mAbs that neutralize EV-A71 were purified, those with VkI, VkIII and VkIV were purified by using protein L resin (Genescript, L00239), the others were purified by mixed-mode chromatography and anion-exchange chromatography as described previously [51].

### Neutralization assays

Neutralizing antibody titers against EV-A71 in serum samples from patients and cell culture supernatant containing mAbs or purified mAbs were measured using two EV-A71 strains, a subgenotype C4a strain (GenBank, EU703812.1), the major EV-A71 genotypes circulating in mainland China since 2007, and a C4b strain (GenBank, HM064456.1). Neutralization assays were performed with RD cells (ATCC, CCL-136) maintained in Dulbecco’s modified Eagle’s medium (Gibco, 11965118). The titer of virus stock was determined by titration in RD cells. The 50% tissue culture infectious dose (TCID_50_) was calculated using the Spearman-Karber method. For the neutralization assay, samples were heat inactivated at 56°C for 30 min, and twofold serial diluted. The diluted sample were incubated with 100 TCID_50_/50μl virus in 96-well plates at 37°C for two hours and then 1E4/100μl RD cells were added. The plate was incubated at 37°C in 5% CO_2_ for 5–7 days. Cytopathic effect was observed by microscopy. Serum samples and mAbs were tested in duplicates. Positive and negative serum controls and back titration were performed in each batch of test. Antibody titers were defined as the reciprocal of the highest dilution of each serum capable of inhibiting 50% of the cytopathic effect and calculated using the Spearman-Karber method.

In the pre-attachment neutralization assay, serially diluted mAbs were incubated with 100 TCID_50_ of EV-A71 at 37°C for 1 hour. The mixtures were then chilled to 4°C and added to RD cells plated the day before and incubated at 4°C for 1 hour. After incubation, the cells were washed to remove unbound viruses. The plates were then incubated at 37°C in 5% CO_2_ for 5 days. In the post-attachment neutralization assay, 100 TCID_50_ of chilled EV-A71 was added into plated RD cells and incubated at 4°C for 1 hour. The cells were then washed to remove unbound viruses and serial diluted mAbs were added and incubated at 37°C for 1 hour. The cells were washed again and then incubated at 37°C in 5% CO_2_ for 5 days. At the end of incubation, the viability of cells was determined by using Cell Counting Kit-8 (DOJINDO Laboratories, CK04) according to the manufacturer’s instructions.

### Enzyme linked immunosorbent assay

Enzyme linked immunosorbent assay (ELISA) was used to test the binding of mAbs to EV-A71. EV-A71 virions were purified as previously described [33]. The purified mature EV-A71 virions were coated in 96 well high bind microplate (Corning, 9018) at 4°C overnight with 0.4μg per well of virus suspended in 50 mM carbonate buffer (pH 9.4). Non-specific binding was blocked with 5% skimmed milk in PBS. Serial dilutions of cell culture supernatant containing mAbs or purified mAbs were added to the plates. The bound virus-specific antibodies were detected with an HRP-conjugated goat anti-Human IgG, IgM, IgA (H+L) secondary antibody (ThermoFisher Scientific, A18848) followed by colourimetric development using TMB-ELISA Substrate Solution (ThermoFisher Scientific, 34022) and optical density absorbance measured at 450 nm.

In the competition ELISA, EV-A71 pre-coated plates were blocked and incubated with purified IgM mAbs or isotype control. Recombinant SCARB2-hFc or PSGL-1-hFc fused with the Fc region of human IgG1 (Sino Biological) was then added. The bound SCARB2-hFc and PSGL-1-hFc was detected with biotinylated goat anti-human IgG Fc antibody and HRP-streptavidin conjugate. The percent reduction of receptor binding was calculated.

### Selection of escape variants

EV-A71 variants were selected with mAbs as previously described [17]. In brief, plaque-purified wild-type EV-A71 were diluted to a 50 TCID_50_ multiply neutralization titer against 32 μg/ml mAb and incubated with an equal volume of mAb at a final concentration of 32 μg/ml for 1 hour at room temperature. After incubation, the mixture was added into RD cells and incubated for 4 days. Cells and supernatant were freeze-thawed three times, centrifugated to obtain virus containing supernatant. The virus was then plaque purified. The plaque purified escape variants were verified using neutralization assays, the P1 region was sequenced and compared with the sequence of the wild-type strain.

### Statistics

EC50 (half maximal effective concentration) and IC50 (Half-maximal inhibitory concentrations) values were calculated using GraphPad Prism 7 software. To calculate the approximate EC50/IC50 values for poor binding/neutralizing antibodies (such as M2-10 in Fig 2 and post-attachment neutralization of M1-24 and M3-7 in Fig 4), the upper limits of the curves were set to the highest values in the same batch of assay. The Wilcoxon signed rank test was used to compare log2 antibody titers of wild-type with escape variant strains for all samples tested. The ratio of neutralizing antibody titer against variant vs. wild-type was calculated using raw data and compared between mild HFMD patients and severe HFMD patients using the Wilcoxon rank sum test. Spearman’s correlation was used to test the association between age of patients and the ratio of neutralizing antibody titer against variant/wild-type strains.

## Supporting information

S1 Fig Sera neutralizing antibody titers against EV-A71 C4a of six study subjects. Gray dashed line shows threshold for seropositive titer (neutralizing antibody titer ≥ 16).
(TIF)

S2 Fig Uniform Manifold Approximation and Projection (UMAP) plot of PBMC clusters and cluster markers. (A) UMAP and clustering for all PBMCs. (B) Dot plot of markers of PBMC clusters. (C) Sub-clustering of B cells. (D) Dot plot of B cell sub-clusters.
(TIF)

S3 Fig Subclasses and variable gene usage of antibodies in plasmablasts. Numbers (A) and percentages (B) of IgM, IgA, and IgG antibodies in plasmablasts from each study subject. Antibody heavy chain variable gene usage in plasmablasts from mild HFMD patients (C) and severe HFMD patients (D), the values of each plot sum up to 100%.
(TIF)

S4 Fig Schematic diagram of plasmablast selection and antibody screening. (TIF)

S5 Fig Percentages of heavy chain variable gene usage of EV-A71 specific mAbs and EV-A71 neutralizing mAbs identified in this study. Variable gene usage was determined using IMGT/V-Quest tool.
(TIF)

S6 Fig Neutralization of EV-A71 infection at the pre-attachment and post-attachment stages by IgG1 and IgM mAbs. Inhibition of EV-A71 (C4a) infection at pre-attachment (red) and post-attachment (black) stages were determined by neutralization assay, cell viability was determined using Cell Counting Kit-8. (A) mAbs neutralizing EV-A71 at post-attachment stage at much higher concentrations than at pre-attachment stage. (B) mAb neutralized EV-A71 at similar concentrations at two stages. The curves were fit by nonlinear regression. Half-maximal inhibitory concentrations (IC50) were calculated using GraphPad Prism 7 software. (C) Receptor competition of IgM mAbs were tested by using ELISA assay. Plates pre-coated with purified mature EV-A71 virion were incubated with mAbs and then recombinant receptors were added. Numbers indicate the percent reduction of binding of receptors.
(TIF)

S7 Fig Alignment of P1 region of major EV-A71 genotypes. Amino acid sequences of representative strains of genotypes and subgenotypes of EV-A71 were aligned. Residues vary among these strains are shown. The six residues critical for neutralization activity of mAbs identified in this study are conserved among EV-A71 genotypes.
(TIF)

S8 Fig Epitopes critical for activity of human EV-A71 neutralizing mAb combining the data of this study and data from Huang et al. (A) Neutralizing antibodies reported by Huang et al. were used for selection of EV-A71 C4a and EV-A71 C4b escape variants. (B) Mapping of residues critical for neutralization of mAbs in this study and those from Huang et al.
(TIF)

S9 Fig Serum antibody titers against wild-type and variant EV-A71 strains were compared between severe HFMD patients and mild HFMD patients. A to J, neutralizing titer of serum samples against wild-type EV-A71 and variant strains, as grouped by disease severity. K to T, comparison of fold changes in neutralizing titers against wild-type EV-A71 and variant strains between patients with mild and severe HFMD. The ratio of neutralizing antibody titer against variant vs. wild-type was calculated using raw data and compared between patients with mild and severe HFMD using the Wilcoxon rank sum test.
(TIF)

S10 Fig Correlation between patient age and fold-change of neutralization titers against each escape variant compared with wild-type EV-A71. (TIF)

S1 Table Demographic and clinical characteristics of the study subjects. (DOCX)

S2 Table Non-neutralizing EV-A71 binding IgG1 mAbs. (DOCX)

S3 Table Summary of mAbs screened for each patient. (DOCX)

S4 Table Variable region sequences of 145 mAbs produced in this study. (XLSX)

S5 Table Demographic and clinical characteristics of patients included in tests of epitope specific antibody responses. (DOCX)
