# Supplementary figures and images for: Antibody signatures in hospitalized hand, foot and mouth disease patients with acute enterovirus A71 infection
Source: PLoS Pathog. 2023 Jun 1;19(6):e1011420. doi: 10.1371/journal.ppat.1011420 (PMC10263328; doi:10.1371/journal.ppat.1011420)

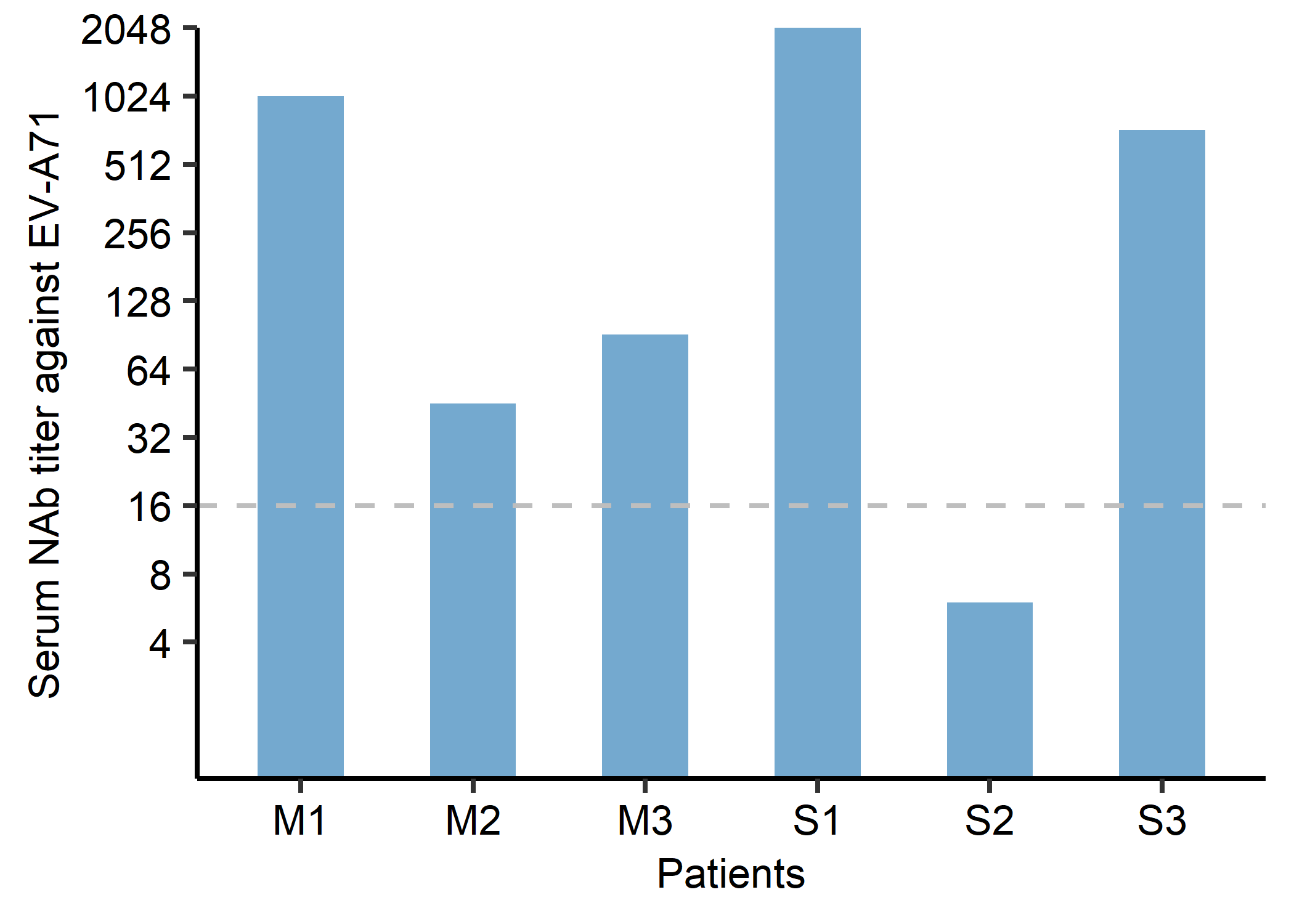

Supplement: S1 Fig — Gray dashed line shows threshold for seropositive titer (neutralizing antibody titer ≥ 16). (TIF) [file ppat.1011420.s001.tif]

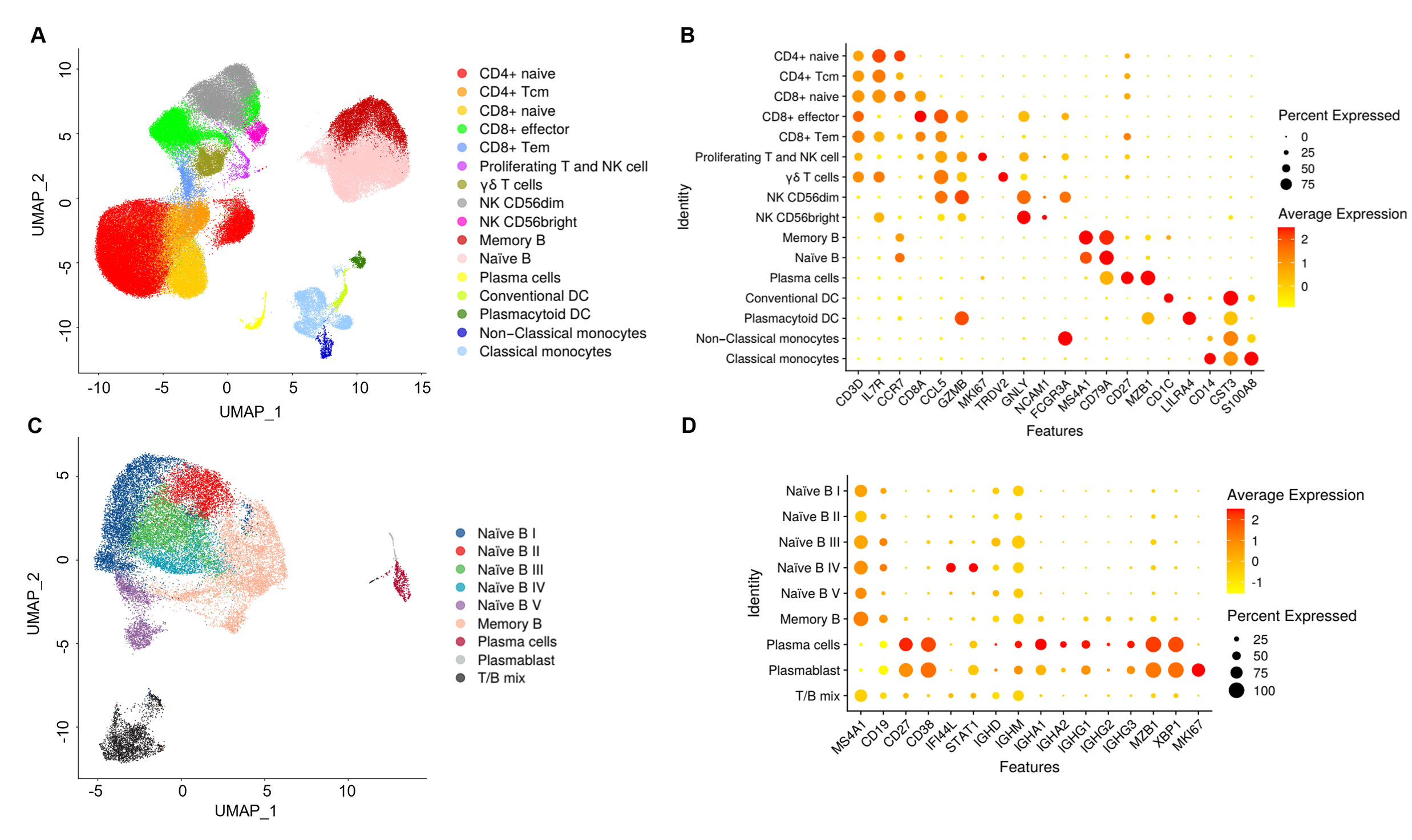

Supplement: S2 Fig — (A) UMAP and clustering for all PBMCs. (B) Dot plot of markers of PBMC clusters. (C) Sub-clustering of B cells. (D) Dot plot of B cell sub-clusters. (TIF) [file ppat.1011420.s002.tif]

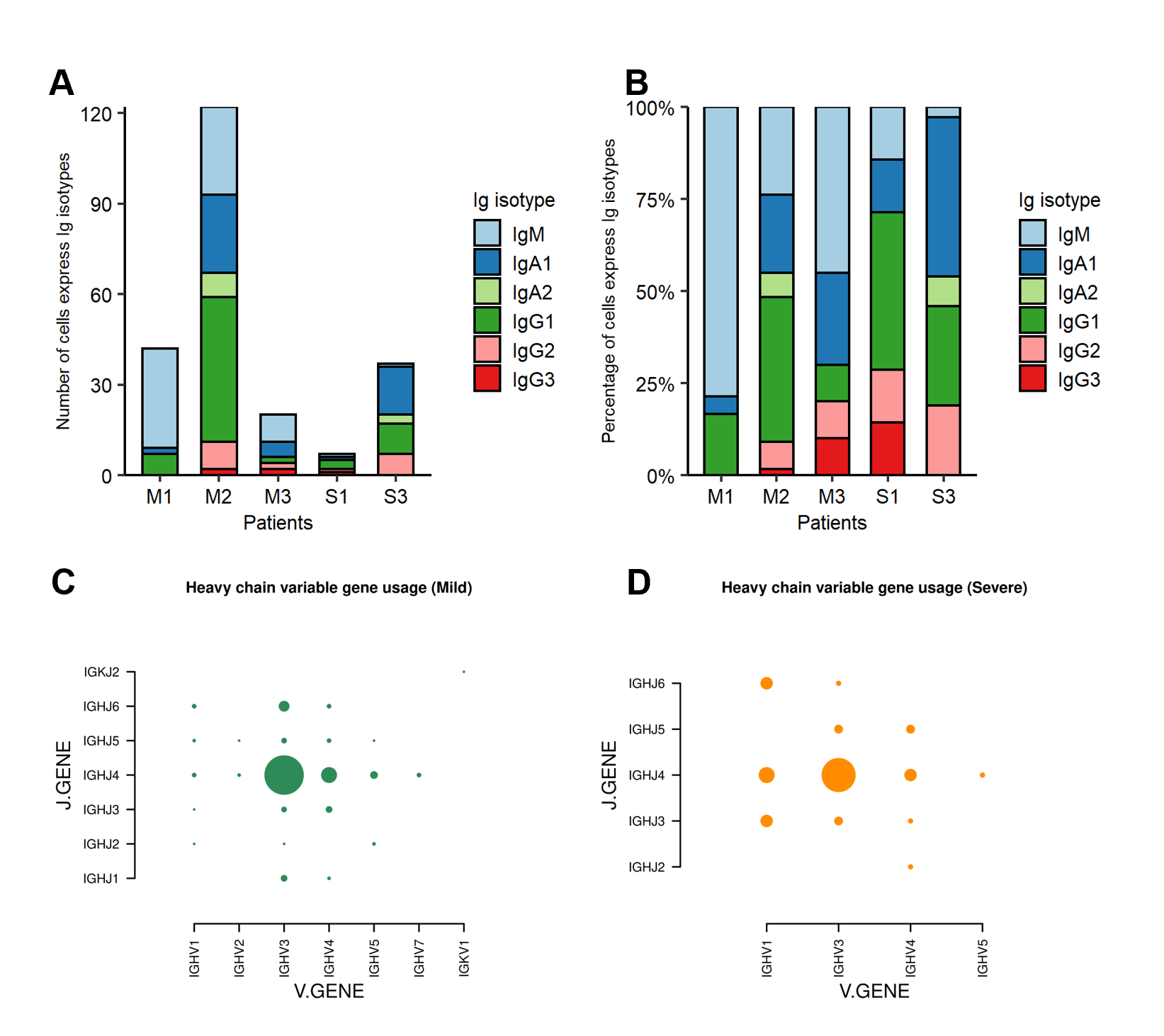

Supplement: S3 Fig — Numbers (A) and percentages (B) of IgM, IgA, and IgG antibodies in plasmablasts from each study subject. Antibody heavy chain variable gene usage in plasmablasts from mild HFMD patients (C) and severe HFMD patients (D), the values of each plot sum up to 100%. (TIF) [file ppat.1011420.s003.tif]

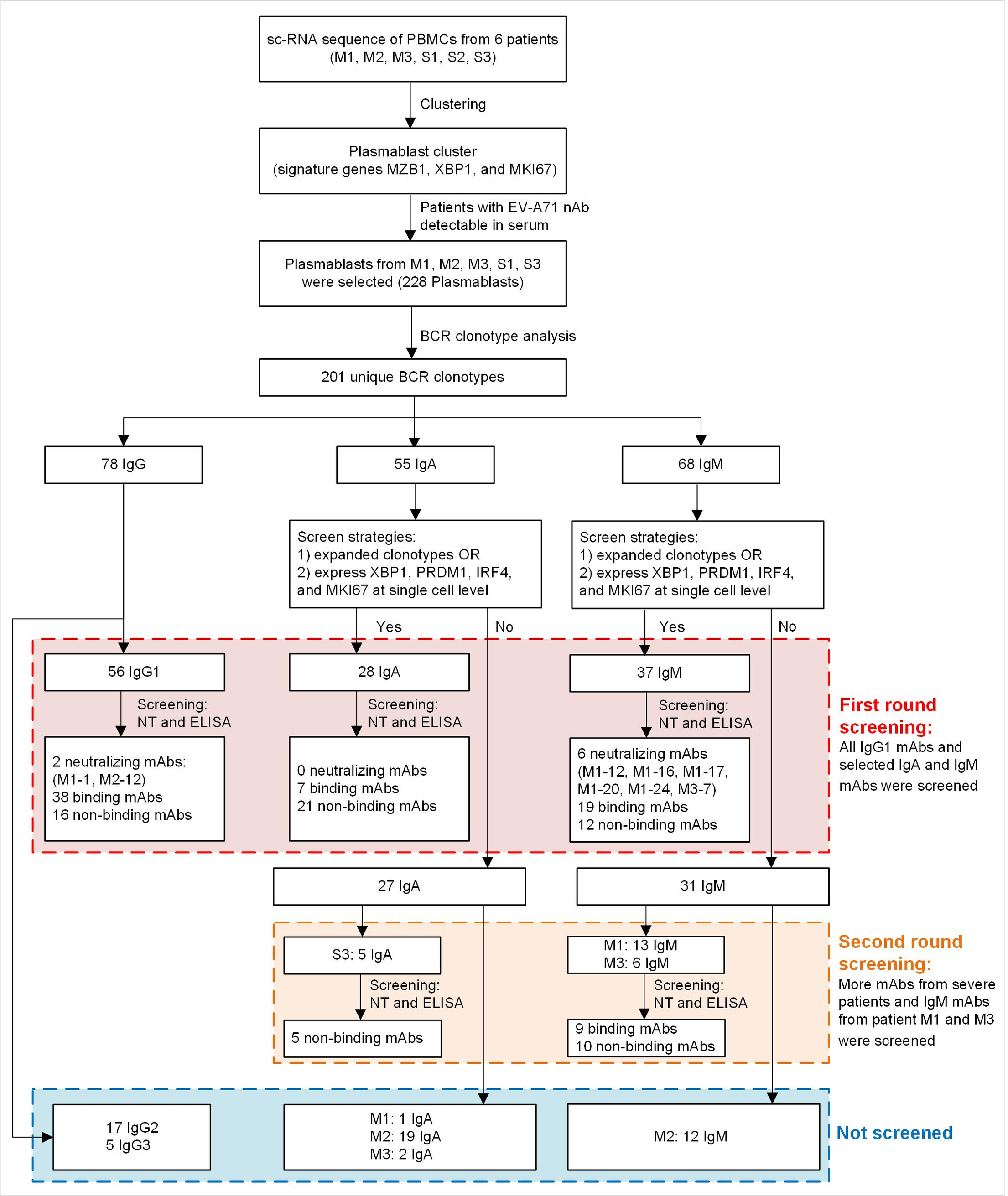

Supplement: S4 Fig — (TIF) [file ppat.1011420.s004.tif]

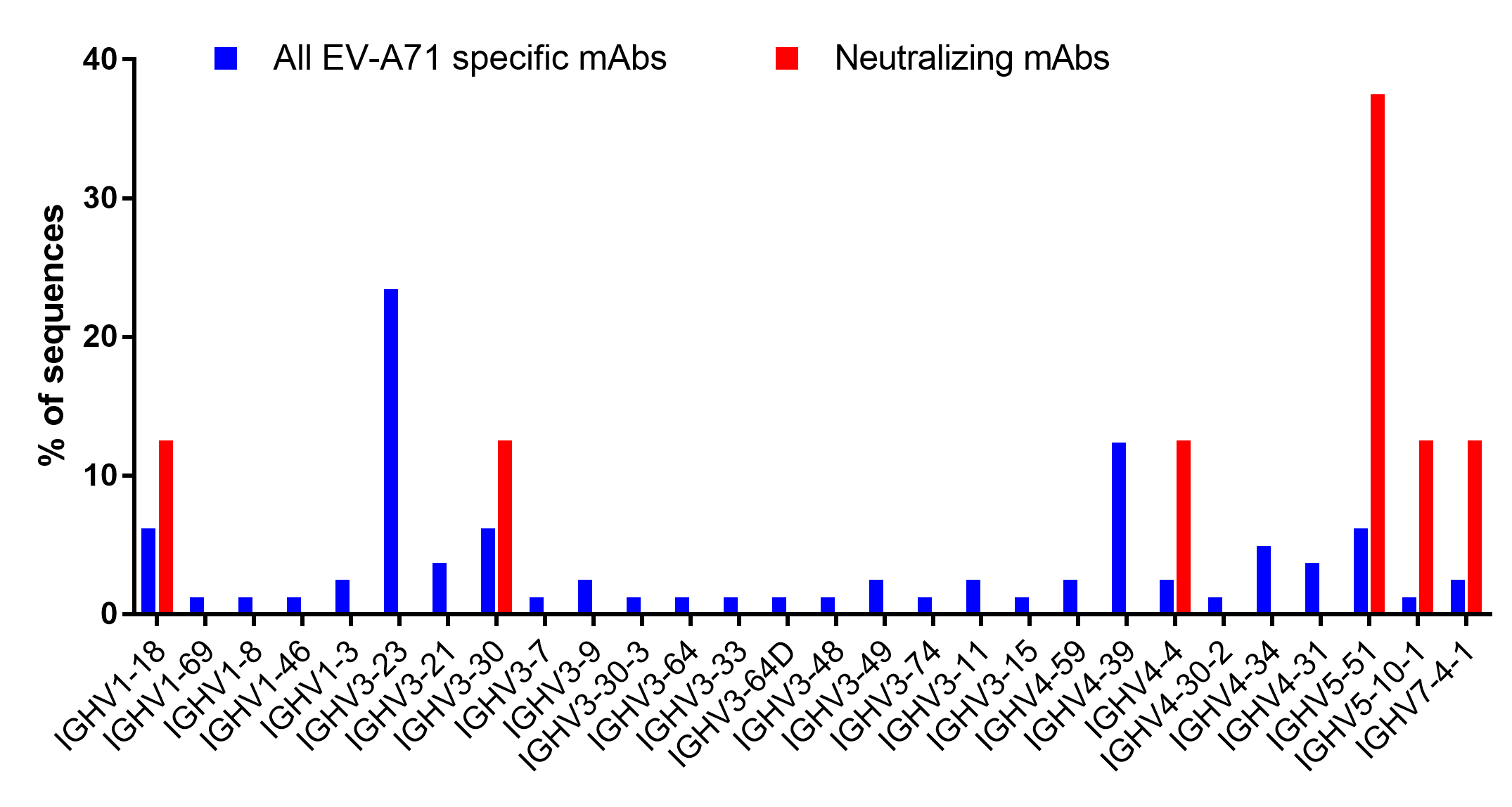

Supplement: S5 Fig — Variable gene usage was determined using IMGT/V-Quest tool. (TIF) [file ppat.1011420.s005.tif]

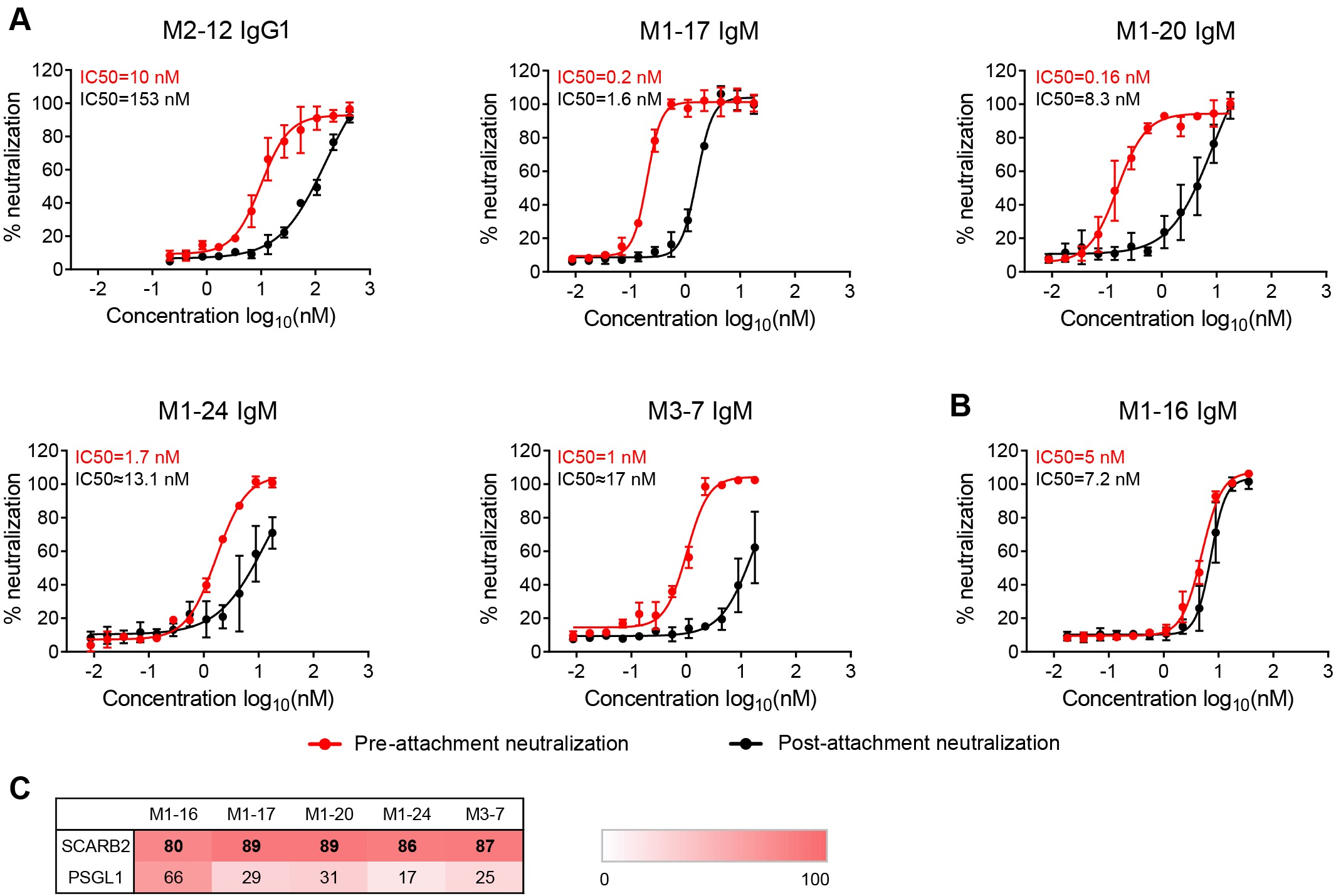

Supplement: S6 Fig — Inhibition of EV-A71 (C4a) infection at pre-attachment (red) and post-attachment (black) stages were determined by neutralization assay, cell viability was determined using Cell Counting Kit-8. (A) mAbs neutralizing EV-A71 at post-attachment stage at much higher concentrations than at pre-attachment stage. (B) mAb neutralized EV-A71 at similar concentrations at two stages. The curves were fit by nonlinear regression. Half-maximal inhibitory concentrations (IC50) were calculated using GraphPad Prism 7 software. (C) Receptor competition of IgM mAbs were tested by using ELISA assay. Plates pre-coated with purified mature EV-A71 virion were incubated with mAbs and then recombinant receptors were added. Numbers indicate the percent reduction of binding of receptors. (TIF) [file ppat.1011420.s006.tif]

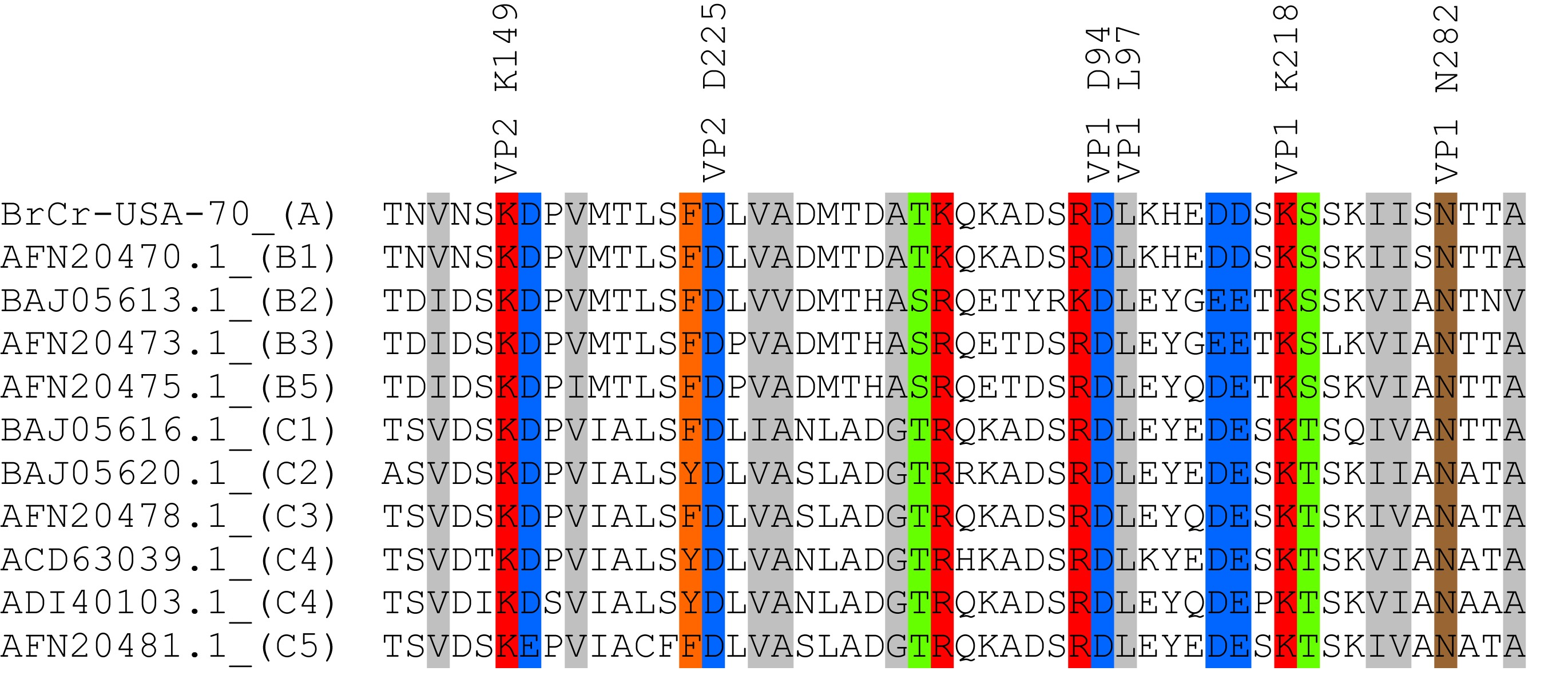

Supplement: S7 Fig — Amino acid sequences of representative strains of genotypes and subgenotypes of EV-A71 were aligned. Residues vary among these strains are shown. The six residues critical for neutralization activity of mAbs identified in this study are conserved among EV-A71 genotypes. (TIF) [file ppat.1011420.s007.tif]

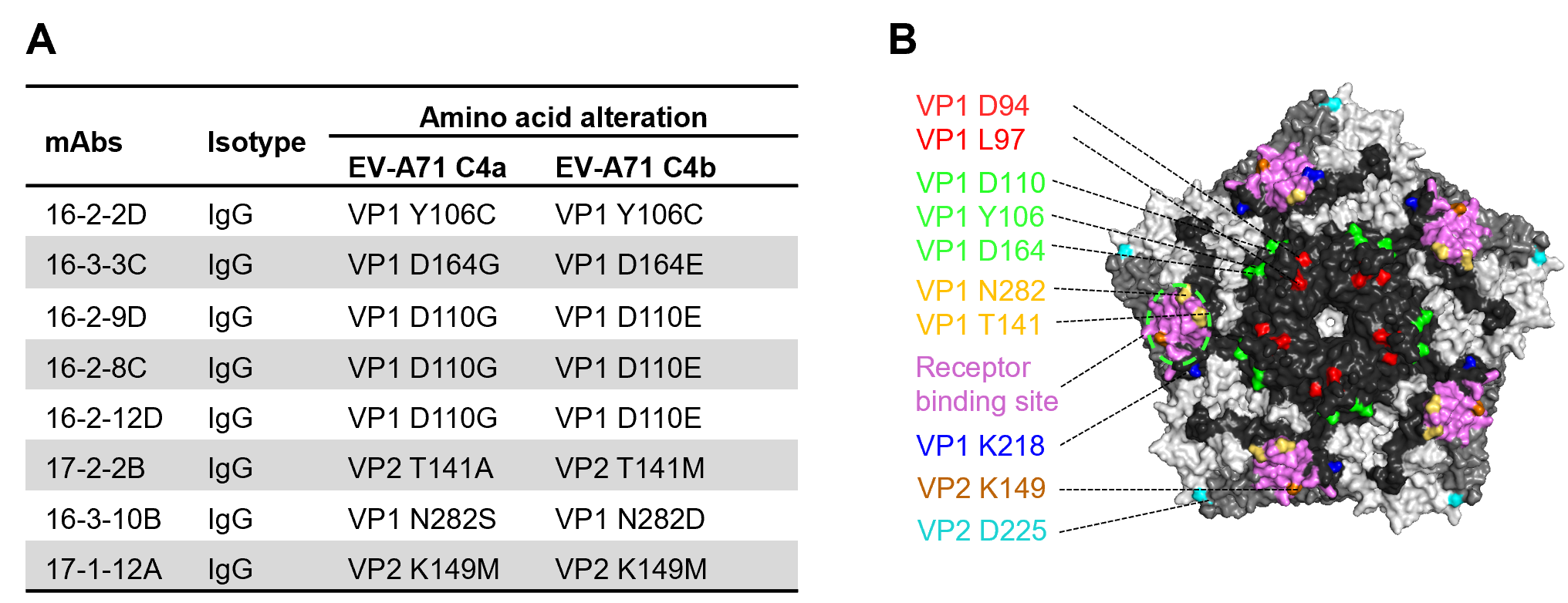

Supplement: S8 Fig — (A) Neutralizing antibodies reported by Huang et al. were used for selection of EV-A71 C4a and EV-A71 C4b escape variants. (B) Mapping of residues critical for neutralization of mAbs in this study and those from Huang et al. (TIF) [file ppat.1011420.s008.tif]

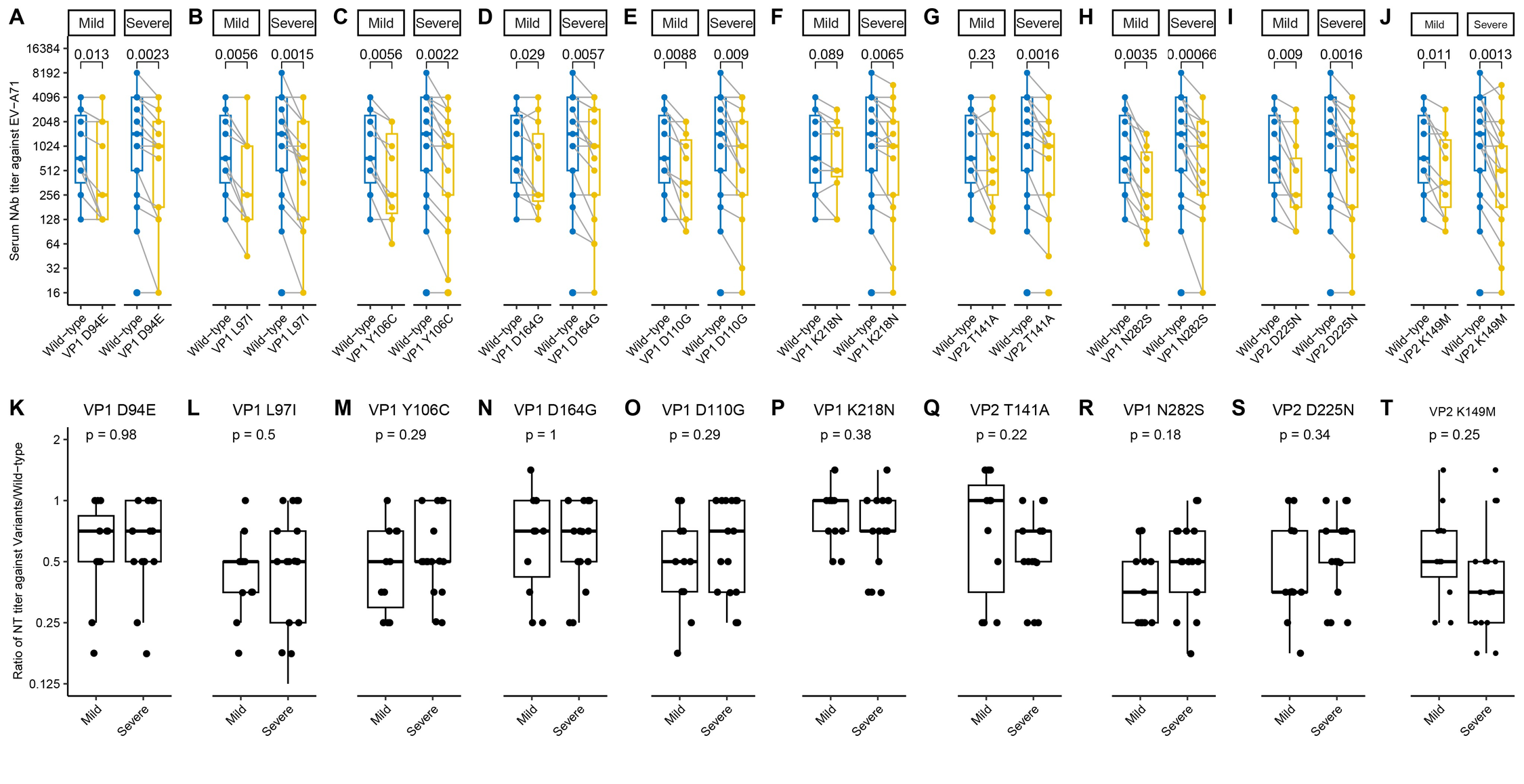

Supplement: S9 Fig — A to J, neutralizing titer of serum samples against wild-type EV-A71 and variant strains, as grouped by disease severity. K to T, comparison of fold changes in neutralizing titers against wild-type EV-A71 and variant strains between patients with mild and severe HFMD. The ratio of neutralizing antibody titer against variant vs. wild-type was calculated using raw data and compared between patients with mild and severe HFMD using the Wilcoxon rank sum test. (TIF) [file ppat.1011420.s009.tif]

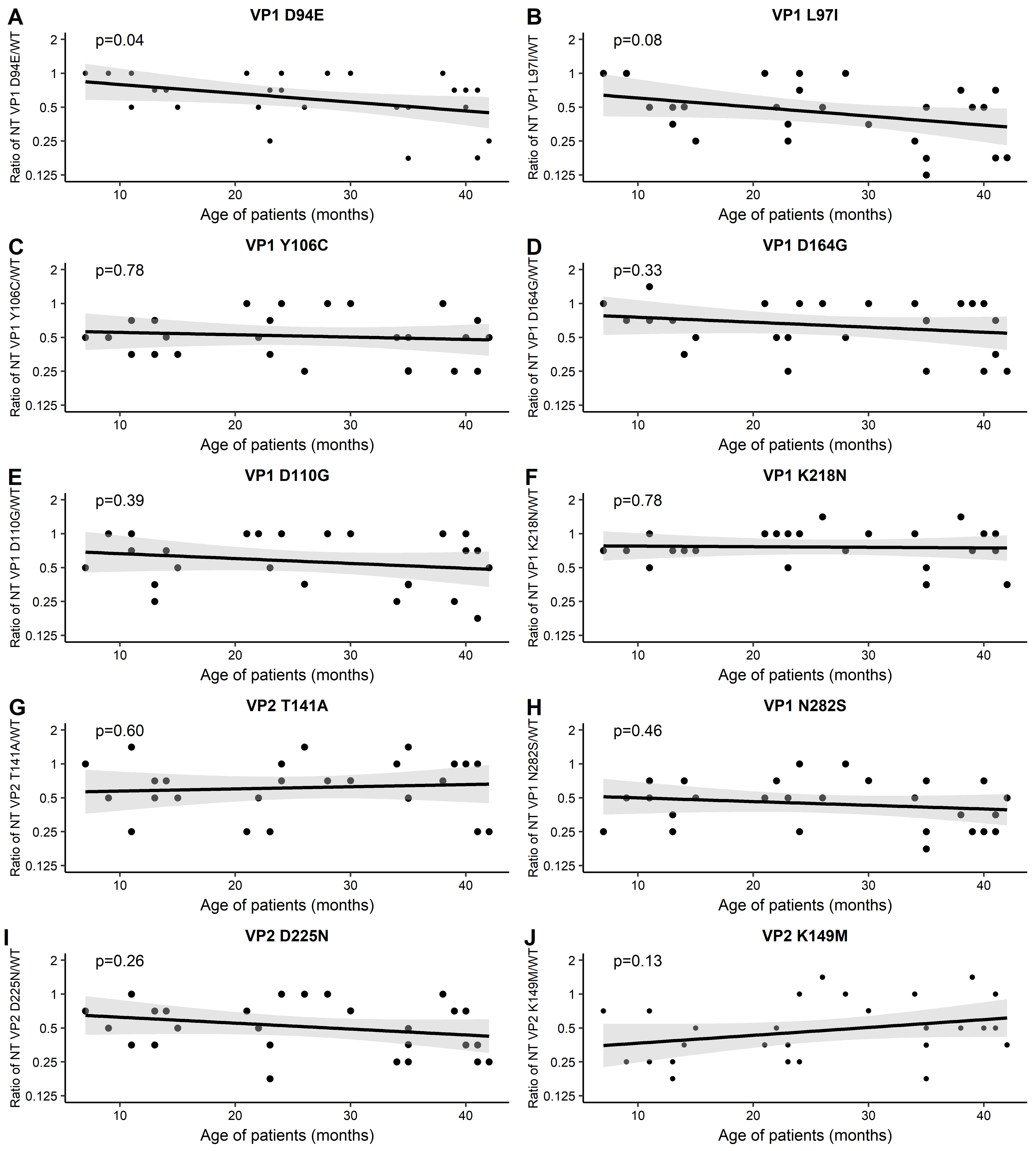

Supplement: S10 Fig — (TIF) [file ppat.1011420.s010.tif]
