# Supplementary material for: Antibody signatures in hospitalized hand, foot and mouth disease patients with acute enterovirus A71 infection
Source: PLoS Pathog. 2023 Jun 1;19(6):e1011420. doi: 10.1371/journal.ppat.1011420 (PMC10263328; doi:10.1371/journal.ppat.1011420)
Supplement: S1 Table — (DOCX) [file ppat.1011420.s011.docx]

**S1 Table. Demographic and clinical characteristics of the study subjects.**

| **Patient ID** | **M1** | **M2** | **M3** | **S1** | **S2** | **S3** |
| --- | --- | --- | --- | --- | --- | --- |
| **Gender** | M | F | M | M | M | F |
| **Age (months)** | 7 | 26 | 35 | 9 | 24 | 34 |
| **Severity** | Mild HFMD | Mild HFMD | Mild HFMD | Severe HFMD | Severe HFMD | Severe HFMD |
| **Complications** | None | None | None | Brainstem encephalitis | Brainstem encephalitis | Brainstem encephalitis |
| **ICU admission** | No | No | No | Yes | Yes | Yes |
| **Length of Stay, days** | 5 | 5 | 4 | 13 | 12 | 12 |
| **Sampling time^a^** | 3 | 1 | 4 | 5 | 1 | 5 |

^a^ days from illness onset. HFMD, hand, foot and mouth disease; ICU, intensive care unit.
