# Supplementary material for: Antibody signatures in hospitalized hand, foot and mouth disease patients with acute enterovirus A71 infection
Source: PLoS Pathog. 2023 Jun 1;19(6):e1011420. doi: 10.1371/journal.ppat.1011420 (PMC10263328; doi:10.1371/journal.ppat.1011420)
Supplement: S2 Table — (DOCX) [file ppat.1011420.s012.docx]

**S2 Table. Non-neutralizing EV-A71 binding IgG1 mAbs.**

| **mAb** | **Isotype** | **V_H_** | **D_H_** | **J_H_** | **Heavy-chain junction sequence** | **Mutations** | **V_L_** | **J_L_** | **Light-chain junction sequence** | **Mutations** | **EC_50_(μg/ml) C4a** |
| --- | --- | --- | --- | --- | --- | --- | --- | --- | --- | --- | --- |
| M1-2 | IgG1 | 3-30*03 or 18 | 3-22*01 | 4*02 | CAKEYYYDSSAYADYW | 1 (1) | 1-8*01 | 1*01 | CQQYYSYPWTF | 2 (1) | 4.5 |
| M1-5 | IgG1 | 4-34*01 | 5-18*01 | 6*03 | CARAHRYGLLGNLYYYMDVW | 10 (7) | 1-44*01 | 1*01 | CAAWDDSLNGPVF | 4 (4) | 19.9 |
| M1-6 | IgG1 | 4-34*01 | 6-19*01 | 4*02 | CARVRINIPLYSSGWPGSNSYIDYW | 2 (2) | 1-39*01 | 2*01 | CQQSYSTPYTF | 4 (4) | 3.734 |
| M2-10 | IgG1 | 4-59*08 | 1-1*01 | 3*02 | CARLRRQRDAFDIW | 10 (6) | 3-20*01 | 2*01 | CQQYGSSTGYTF | 2 (2) | 18.5 |
| M2-11 | IgG1 | 4-39*01 | 2-8*01 | 4*02 | CARRKLSRGVLLVYAPFDYW | 7 (5) | 3-21*04 | 2*01, or 3*01 | CQVWDSGSDLVVF | 9 (6) | 21.67 |
| M2-13 | IgG1 | 4-39*01 | 6-19*01 | 4*02 | CARRPALALTGTGRLTFDYW | 21 (13) | 1-5*04 | 1*01 | CQQYDSYPWTF | 17 (12) | 27.9 |
| M2-15 | IgG1 | 3-23*01 | 3-16*02 | 4*02 | CAKNDYVWGSYPFDYW | 4 (3) | 1D-16*01 | 4*01 | CQQYNSYPLTF | 1 (0) | 5 |
| M2-16 | IgG1 | 1-18*01 | 3-22*01 | 6*03 | CARGLDQQFYYDSTGRRRTYFSYYMDVW | 13 (5) | 8-61*01 | 2*01 | CALYMGSGIWLF | 24 (13) | 5.9 |
| M2-18 | IgG1 | 1-3*01 | 5-18*01 | 5*01 | CARRPARGGYSNGYDYW | 9 (6) | 2-28*01 | 3*01 | CMQALQTPPTF | 4 (2) | 6.9 |
| M2-19 | IgG1 | 3-23*01 | 3-3*02 | 4*02 | CAKELHFLTGYRWGSQPDYTFDSW | 26 (16) | 2-14*03 | 1*01 | CSSYTTSSTLVF | 17 (11) | 5.18 |
| M2-2 | IgG1 | 3-48*03 | 3-10*01 | 5*02 | CARINVVRGVIMTRGWFDPW | 12 (8) | 3-21*04 | 1*01 | CQVWDSSSDHYVF | 8 (5) | 3.559 |
| M2-20 | IgG1 | 3-30*04 | 3-3*01 | 5*02 | CAGVGVDPW | 23 (12) | 1-27*01 | 1*01 | CQKYNSAPWTF | 11 (8) | 17.7 |
| M2-21 | IgG1 | 4-31*03 | 4-23*01 | 4*02 | CARGKRSEDYGGNSIFDSW | 21 (14) | 4-1*03 | 2*01 | CHQYYTIPHTF | 10 (8) | 14.2 |
| M2-22 | IgG1 | 3-23*01 | 3-3*01 | 4*02 | CAKDRVSTYYDFWSGYYDRPNFDYW | 0 (0) | 1-47*01 | 3*02 | CASWDDSLSGPVF | 1 (1) | 11 |
| M2-23 | IgG1 | 5-51*01 | 2-2*01 | 2*01 | CARLSPEYCSGNSCLYWYFDLW | 18 (13) | 3-25*03 | 3*02 | CQSADSSGFHWLF | 17 (11) | 18 |
| M2-24 | IgG1 | 3-9*01 | 3-9*01 | 6*03 | CAKAPAKYSFLNYMDVW | 14 (7) | 3-15*01 | 4*01 | CQQYNNWPPLTF | 9 (5) | 7.8 |
| M2-25 | IgG1 | 3-49*04 | 3-16*02 | 3*02 | CTRGRVWGSHRYHAFDIW | 11 (8) | 3-11*01 | 4*01 | CQQRSAWPLTF | 6 (4) | 7.1 |
| M2-26 | IgG1 | 4-39*01 | 3-22*01 | 4*02 | CARRLYYDYSGFDYW | 17 (8) | 1-39*01 | 2*01 | CQQSYSRPYTF | 17 (8) | 8.2 |
| M2-27 | IgG1 | 4-31*03 | 2-21*02 | 4*02 | CTRTPGRVDNFSDYW | 14 (10) | 1-16*01 | 1*01 | CQHYSTYPPTF | 17 (12) | 22 |
| M2-28 | IgG1 | 3-74*01 | 6-13*01 | 6*02 | CARQQQLADYYYYGMDVW | 0 (0) | 4-1*01 | 4*01 | CQQYYSTPLTF | 0 (0) | 12 |
| M2-29 | IgG1 | 3-11*06 | 3-22*01 | 4*02 | CVRDRSSVKYYYDNSAYWLFDSW | 9 (7) | 3-19*01 | 1*01 | CSSRDNSGNLYVF | 7 (5) | 15 |
| M2-32 | IgG1 | 1-18*01 | 3-10*01 | 6*02 | CARVRGAMVRGVIITSAYYGMDVW | 0 (0) | 1D-16*01 | 4*01 | CQQYNSYPLTF | 0 (0) | 18.5 |
| M2-33 | IgG1 | 3-49*04 | 6-13*01 | 4*02 | CTRPLYTSSWVDYFDSW | 10 (9) | 2-14*03 | 1*01 | CSSYRSDSTYVF | 4 (3) | 8.6 |
| M2-34 | IgG1 | 4-59*08 | 1-26*01 | 4*02 | CARRKWELQLDYW | 11 (6) | 1-39*01 | 2*01 | CQQSYSTPYTF | 26 (10) | 31 |
| M2-35 | IgG1 | 3-11*01 | 3-22*01 | 3*02 | CARDILPRQDYYNSSPDAFDIW | 5 (4) | 2-28*01 | 2*01 | CMQALQTPYTF | 0 (0) | 5.9 |
| M2-6 | IgG1 | 4-34*01 | 3-16*01 | 5*02 | CARRGMTYVHLDNWFDPW | 0 (0) | 3-19*01 | 3*02 | CNSRDSSGNHWVF | 0 (0) | 12.6 |
| M2-7 | IgG1 | 3-21*01 | 1-14*01 | 3*02 | CARDGLLPGPGEAFDIW | 0 (0) | 1-44*01 | 3*02 | CAAWDDSLNGWVF | 0 (0) | 29.6 |
| M2-8 | IgG1 | 3-23*01 | 3-3*01 | 4*02 | CAKDSPRRITIFSVVIHYFDLW | 15 (8) | 1-27*02 | 5*01 | CQNYYSAPITF | 6 (4) | 14 |
| M2-9 | IgG1 | 3-21*01 | 3-22*01 | 6*03 | CARDATKYYYDSSDFYRNNCMDVW | 5 (4) | 1-39*01 | 4*01 | CQQSYSTPLTF | 12 (8) | 6.7 |
| S1-1 | IgG1 | 3-9*01 | 2-8*01 | 3*02 | CARDSLGSERWNALDIW | 12 (8) | 4-1*01 | 1*01 | CQQYYDAPRTF | 8 (5) | 4.14 |
| S1-2 | IgG1 | 1-69*01 | 3-10*01 | 3*02 | CAREHLYGSGYYSLTLNAFDIW | 19 (15) | 2D-29*01 | 4*01 | CMQSIQLPLSF | 12 (7) | 12.3 |
| S3-1 | IgG1 | 3-15*01 | 5-12*01 | 4*02 | CTTAGYGGFDDDFW | 16 (10) | 2-30*01 | 5*01 | CMQATHWPPGTF | 7 (6) | 11.1 |
| S3-2 | IgG1 | 4-34*01 | 4-11*01 | 5*02 | CARGGTTFTDPW | 1 (1) | 3-15*01 | 2*01 | CQQYKNWPPYTF | 5 (5) | 17.4 |
| S3-3 | IgG1 | 1-18*01 | 2-2*01 | 4*02 | CARANVVVPAAIVYPIRSLRFFDYW | 14 (9) | 2-14*03 | 2*01, or 3*01 | CTSCSTSDTVAVVF | 17 (14) | 16.7 |
| S3-5 | IgG1 | 1-18*04 | 2-2*01 | 3*01 or 02 | CARDRGVVVSAALLYHFWRMHPYDLW | 32 (17) | 4-1*01 | 1*01 | CQQYYDTLRTF | 15 (10) | 6.248 |
| S3-7 | IgG1 | 4-4*07 | 2-2*01 | 5*02 | CARTHIVLLPAATPHGWFDPW | 7 (3) | 1-39*01 | 1*01 | CQQSYSAPQWTF | 3 (2) | 26.14 |
| S3-8 | IgG1 | 3-33*01 or 06 | 6-13*01 | 4*02 | CAKSYADIGGSWYNADFW | 10 (6) | 2-11*01 | 3*02 | CCSYAASYTWVF | 4 (3) | 8.9 |
| S3-9 | IgG1 | 1-46*01 or 03 | 2-2*01 | 6*02 | CARDRRPVPAATRGLDVW | 11 (6) | 2-14*03 | 3*02 | CSSYTTRSSWVF | 7 (6) | 7.3 |

V_H_, variable gene segment of the heavy-chain variable domain; D_H_, diversity gene segment of the heavy-chain variable domain; J_H_, joining gene segment of the heavy-chain variable domain; Mutations, mutation number of variable domain nucleotides (amino acids); V_L_, variable gene segment of the light-chain variable domain; J_L_, joining gene segment of the light-chain variable domain.
