# Supplementary material for: Antibody signatures in hospitalized hand, foot and mouth disease patients with acute enterovirus A71 infection
Source: PLoS Pathog. 2023 Jun 1;19(6):e1011420. doi: 10.1371/journal.ppat.1011420 (PMC10263328; doi:10.1371/journal.ppat.1011420)
Supplement: S3 Table — (DOCX) [file ppat.1011420.s013.docx]

**S3 Table.** **Summary of mAbs screened for each patient.**

| **Patient ID** | | | **M1** | **M2** | **M3** | **S1** | **S3** | **Sum** |
| --- | --- | --- | --- | --- | --- | --- | --- | --- |
| Total No. of plasmablast BCR clonotypes | | | 42 | 100 | 20 | 7 | 32 | 201 |
| No. of IgG clonotypes | | | 7 | 46 | 6 | 5 | 14 | 78 |
| IgG1 | First round screening | No. of mAb screened | 7 | 35 | 2 | 3 | 9 | **56** |
|  |  | No. of neutralizing mAbs | 1 | 1 | 0 | 0 | 0 | 2 |
|  |  | No. of binding mAbs | 3 | 26 | 0 | 2 | 7 | 38 |
|  |  | No. of non-binding mAbs | 3 | 8 | 2 | 1 | 2 | 16 |
| IgG2 | Not screened | | 0 | 9 | 2 | 1 | 5 | 17 |
| IgG3 | Not screened | | 0 | 2 | 2 | 1 | 0 | 5 |
| IgA | No. of IgA clonotypes | | 2 | 30 | 5 | 1 | 17 | 55 |
|  | First round screening | No. of mAb screened | 1 | 11 | 3 | 1 | 12 | **28** |
|  |  | No. of neutralizing mAbs | 0 | 0 | 0 | 0 | 0 | 0 |
|  |  | No. of binding mAbs | 0 | 3 | 1 | 0 | 3 | 7 |
|  |  | No. of non-binding mAbs | 1 | 8 | 2 | 1 | 9 | 21 |
|  | Second round screening | No. of mAb screened | 0 | 0 | 0 | 0 | 5 | **5** |
|  |  | No. of neutralizing mAbs | \ | \ | \ | \ | 0 | 0 |
|  |  | No. of binding mAbs | \ | \ | \ | \ | 0 | 0 |
|  |  | No. of non-binding mAbs | \ | \ | \ | \ | 5 | 5 |
|  | Not screened | | 1 | 19 | 2 | 0 | 0 | 22 |
| IgM | No. of IgM clonotypes | | 33 | 24 | 9 | 1 | 1 | 68 |
|  | First round screening | No. of mAb screened | 20 | 12 | 3 | 1 | 1 | **37** |
|  |  | No. of neutralizing mAbs | 5 | 0 | 1 | 0 | 0 | 6 |
|  |  | No. of binding mAbs | 12 | 6 | 1 | 0 | 0 | 19 |
|  |  | No. of non-binding mAbs | 3 | 6 | 1 | 1 | 1 | 12 |
|  | Second round screening | No. of mAb screened | 13 | 0 | 6 | 0 | 0 | **19** |
|  |  | No. of neutralizing mAbs | 0 | \ | 0 | \ | \ | 0 |
|  |  | No. of binding mAbs | 8 | \ | 1 | \ | \ | 9 |
|  |  | No. of non-binding mAbs | 5 | \ | 5 | \ | \ | 10 |
|  | Not screened | | 0 | 12 | 0 | 0 | 0 | 12 |

BCR, B cell receptor.
