# Supplementary material for: Antibody signatures in hospitalized hand, foot and mouth disease patients with acute enterovirus A71 infection
Source: PLoS Pathog. 2023 Jun 1;19(6):e1011420. doi: 10.1371/journal.ppat.1011420 (PMC10263328; doi:10.1371/journal.ppat.1011420)
Supplement: S5 Table — (DOCX) [file ppat.1011420.s015.docx]

### S5 Table. Demographic and clinical characteristics of patients included in tests of epitope specific antibody responses.

| Characteristics | Total (N=28) | Mild HFMD patients (n=11) | Severe HFMD patients (n=17) |
| --- | --- | --- | --- |
| Males | 17/28 (61) | 6/11 (55) | 11/17 (65) |
| Age, median months (IQR) | 25 (14-37) | 26 (13-40) | 24 (14-36) |
| Complications |  |  |  |
| Encephalitis | 5/28 (18) | 0/11 (0) | 5/17 (30) |
| Brainstem encephalitis | 10/28 (36) | 0/11 (0) | 10/17 (60) |
| Encephalomyelitis | 2/28 (7) | 0/11 (0) | 2/17 (12) |
| Pulmonary oedema/haemorrhage | 2/28 (7) | 0/11 (0) | 2/17 (12) |
| Cardiorespiratory failure | 1/28 (4) | 0/11 (0) | 1/17 (6) |
| ICU admission |  | 0/11 (0) | 13/17 (76) |
| Length of Stay, days | 6.5 (5-11.5) | 5 (4-5) | 9 (7-13) |
| Sampling time^a^ | 3.5 (2-4.75) | 3 (2-4) | 4 (2-5) |

Data are no. (%) of patients, unless otherwise indicated. ^a^ days from illness onset. IQR, interquartile range. The complications are not mutually exclusive.
